# Transcriptomic profiling of adjuvant colorectal cancer identifies three key prognostic biological processes and a disease specific role for granzyme B

**DOI:** 10.1371/journal.pone.0262198

**Published:** 2021-12-31

**Authors:** Anneleen Daemen, Akshata R. Udyavar, Thomas Sandmann, Congfen Li, Linda J. W. Bosch, William O’Gorman, Yijin Li, Amelia Au-Yeung, Chikara Takahashi, Omar Kabbarah, Richard Bourgon, Priti Hegde, Carlos Bais, Meghna Das Thakur

**Affiliations:** 1 Bioinformatics & Computational Biology, Genentech Inc., South San Francisco, California, United States of America; 2 Oncology Biomarker Development, Genentech Inc., South San Francisco, California, United States of America; 3 OMNI Biomarker Development, Genentech Inc., South San Francisco, California, United States of America; McGill University, CANADA

## Abstract

**Background:**

Colorectal cancer (CRC) is a leading cause of cancer-related deaths, with a 5% 5-year survival rate for metastatic disease, yet with limited therapeutic advancements due to insufficient understanding of and inability to accurately capture high-risk CRC patients who are most likely to recur. We aimed to improve high-risk classification by identifying biological pathways associated with outcome in adjuvant stage II/III CRC.

**Methods and findings:**

We included 1062 patients with stage III or high-risk stage II colon carcinoma from the prospective three-arm randomized phase 3 AVANT trial, and performed expression profiling to identify a prognostic signature. Data from validation cohort GSE39582, The Cancer Genome Atlas, and cell lines were used to further validate the prognostic biology. Our retrospective analysis of the adjuvant AVANT trial uncovered a prognostic signature capturing three biological functions—stromal, proliferative and immune—that outperformed the Consensus Molecular Subtypes (CMS) and recurrence prediction signatures like Oncotype Dx in an independent cohort. Importantly, within the immune component, high granzyme B (GZMB) expression had a significant prognostic impact while other individual T-effector genes were less or not prognostic. In addition, we found GZMB to be endogenously expressed in CMS2 tumor cells and to be prognostic in a T cell independent fashion. A limitation of our study is that these results, although robust and derived from a large dataset, still need to be clinically validated in a prospective study.

**Conclusions:**

This work furthers our understanding of the underlying biology that propagates stage II/III CRC disease progression and provides scientific rationale for future high-risk stratification and targeted treatment evaluation in biomarker defined subpopulations of resectable high-risk CRC. Our results also shed light on an alternative GZMB source with context-specific implications on the disease’s unique biology.

## Introduction

Over the past several decades major improvements in outcome for patients with early stage (I, II and III) CRC have been achieved with adjuvant chemotherapy and improved surgical techniques, with 5-year overall survival (OS) ranging from 92% in stage I to 30–70% in stage III [1]. Intervention with chemotherapy in the adjuvant setting has become common practice to improve cure rates for patients with high-risk primary disease. However the method used to classify patients with resectable CRC, the AJCC/UICC-TNM system based on tumor size (T), lymph node spread (N) and metastasis (M) [2–4], has poor specificity in describing prognostic CRC biology and in capturing high-risk CRC patients who recur [5]. This is evidenced by the low number of disease-free survival (DFS) events (182, 17% at 5 years) and OS events (76, 7% at 5 years) in the 1062 biomarker evaluable patients with stage III or high-risk stage II colon carcinoma from the AVANT trial (S1 Fig). Since most stage II and III CRC patients have good prognosis with surgery alone and over 75% of patients with stage II CRC never recur, a sample size of 10,000 patients would be required in a clinical trial to detect a 10% survival benefit from adjuvant therapy with 90% power [6]. This complexity in combination with lack of therapeutic options has prevented changes in the standard of care in this setting. Therefore 3–5 month treatment with oxaliplatin infusion added to Fluorocil and Leucovorin (FOLFOX4) has continued to be common practice over the past decades for surgically resected, stage III or high-risk stage II CRC [7–10]. Thus, a majority of patients who never recur and are not high-risk continue to receive aggressive adjuvant chemotherapy. Furthermore, despite the improvements in patient survival, CRC continues to be a leading cause of cancer-related deaths with more than 500,000 deaths annually, and five-year survival rates in patients with metastatic disease are less than 5% [11].

Several prognostic and recurrence prediction signatures for CRC have been published, such as Oncotype Dx, as well as tumor classification systems with biological interpretability such as the CMS subtypes [12–21], but none have been clinically validated to guide treatment decisions for CRC in the adjuvant setting. Some of these tumor classifications capture tumor-tissue based characteristics, such as the TNM staging system [2–4]. Immunoscore was more recently proposed as a new component of a TNM-Immune classification, based on the observation that the immune contexture of a primary CRC tumor is prognostic [22, 23]. Separate research revealed a negative prognostic role for the stromal compartment as measured by signatures of cancer-associated fibroblasts (CAF) and stromal TGFβ signaling (F-TBRS) [24, 25]. There is a need for a robust and clinically validated assay to more accurately stratify patients with non-metastatic CRC into low- and high-risk groups for more effective management of the disease. Here we present a retrospective analysis of the phase 3 AVANT trial for uncovering the biology of poor prognosis patients in early CRC, and for identifying a *de novo* prognostic signature that captures early CRC patients at high risk of relapse (S2 Fig). This signature uncovered three key biological functions associated with disease progression in high-risk adjuvant CRC patients: (1) proliferative, (2) stromal and (3) immune. Furthermore, we describe a novel and unique role for Granzyme B (GZMB) as a highly prognostic gene in CMS1 and CMS2 CRC, with immune cells being a major source of GZMB in CMS1, while GZMB expression in CMS2 CRC is rather a tumor-intrinsic property, and hence an alternative GZMB source that is worth exploring as a biomarker and to uncover novel biology in CRC.

## Materials and methods

### Study design

AVANT (ClinicalTrials.gov identifier NCT00112918) was a randomized, open-label, prospective, parallel three-arm, phase 3 trial sponsored by F. Hoffmann-La Roche and conducted in accordance with the Declaration of Helsinki. The study design was published previously [7]. Patients (age > = 18 years with histologically confirmed stage III or high-risk stage II colon carcinoma) were subject to surgery with curative intent 4–8 weeks before a 1:1:1 to one of three treatment options: FOLFOX4 for 24 weeks followed by observation for 24 weeks; bevacizumab–FOLFOX4 for 24 weeks followed by bevacizumab monotherapy for 24 weeks; or bevacizumab–XELOX for 24 weeks followed by bevacizumab monotherapy for 24 weeks randomization. Stratification factors included geographic region and disease stage (high-risk stage II vs stage III [N1] vs stage III [N2]). DFS was the primary endpoint and was defined as the time between randomisation and recurrence of the colorectal cancer, new occurrence of colorectal cancer, or death from any cause. From Dec 20, 2004 to June 8, 2007, 3451 patients from 330 centers in 34 countries worldwide were randomly assigned to receive FOLFOX4 (n = 1151), bevacizumab-FOLFOX4 (n = 1155) or bevacizumab-XELOX (n = 1145) [7]. All clinical data are available in S1 Table. All analyses presented herein were done in a retrospective manner and were not prespecified in a prospective analysis plan.

### Sample collection

Baseline formalin-fixed, paraffin-embedded (FFPE) tumor samples were collected from patients who consented to participate in exploratory translational research. Samples with sufficient tissue were selected for further analysis. Of 3451 patients enrolled, 1256 had sufficient material for analysis. Tumors were microdissected to minimize the amount of adjacent normal tissue RNA included in the gene expression analysis. FFPE tissue blocks were sectioned at a thickness of 4 to 6 microns and stored on slides at room temperature. After RNA extraction, samples were stored at -70°C.

### Nanostring gene expression

RNA was extracted from the FFPE patient samples and run on a customized CRC codeset comprised of 829 genes on the Nanostring gene expression platform (NanoString Technologies, Seattle, WA) (S2 Table). The raw probe intensities were corrected for background using blank (water), and then normalized using the NanostringQCPro package in R [26]. Raw counts for 1256 tumor samples were log2 transformed, normalized (common mean and standard deviation for all samples), and gene-wise expression scores were further standardized across all samples by transformation to z-scores. Quality control failures were flagged based on the first principal component of normalized counts; 67 outlier samples were identified. These yielded low overall counts, indicating insufficient input material or another source of assay failure and were removed from further analysis. 12 patients had 2 samples each, all of which were excluded. 1165 samples were deemed biomarker-evaluable samples. For 102 subjects for whom gene expression data was available, no matching clinical annotations were available. The samples from these subjects were excluded from downstream analysis, leaving a final total of 1062 samples. The normalized, z-scored expression data are available in S3 Table. The seven most frequent mutations in codons 12 and 13 of KRAS were assessed with qPCR (12ALA, 12ARG, 12ASP, 12CYS, 12SER, 12VAL, 13ASP). BRAF c.1799T>A (p.V600E) genotyping was performed at Sequenom Laboratories. MSI status was obtained by qPCR, and for our purposes, MSI-low and MSS patients were merged into the MSS category.

### Gene expression based assignment of published CMS subtypes

TCGA RNAseq paired-end data were downloaded from the National Cancer Institute Genomic Data Commons (https://gdc.cancer.gov) and analyzed using HTSeqGenie [27]. KRAS and BRAF mutation information for colon tumors was downloaded from www.cbioportal.org (project Colorectal Adenocarcinoma TCGA provisional) on 09/18/2018. The GSE39582 microarray dataset was downloaded from GEO (GEO accession: GSE39582) [17]. RNAseq data for cell lines were obtained from [28]. The random forest algorithm in the CMS classifier R package [12] was used to assign CMS labels to TCGA and GSE39582 samples based on z-scored log2 transformed gene expression data. Since the AVANT dataset is on a different platform i.e NanoString, we first trained a PAM classifier on the TCGA CRC samples for the prediction of CMS subtype, using expression of the 829 genes on the CRC NanoString panel and the pamr R package. The 132 genes highly predictive of the CMS subtypes in the TCGA subsetted dataset were then used to perform unsupervised hierarchical clustering and tree cutting to annotate the AVANT tumors. For colon cancer cell lines, CMS labels were determined with the nearest-centroid single-sample predictor from the CMS classifier R package [12], assigning a cell line to a CMS in case the correlation to the nearest centroid exceeded 0.15 and the minimal difference between the correlation to the two nearest centroids exceeded 0.6.

### Prognostic signature analysis using elastic net regression

We used elastic net, a regularized regression method that is effective with small sample sizes when the number of features exceeds the number of samples. It uses a grouping approach where collinear or correlated features are represented by one feature and penalizes the remaining collinear features. Using overall survival (OS) data, we built a generalized Cox-regression model using the glmnet R package with alpha values of 0.1–0.9, seed reset in each run, and k-fold cross-validation to reduce overfitting [29, 30]. Lower alpha values result in larger numbers of selected genes. All regression runs provided the same core set of genes that were significantly associated with OS. Additional genes were included for every jump in alpha. As a trade-off between being inclusive of novel biological functions beyond the core set of genes (lower alpha) and a more complex fitted model (higher alpha), we settled on an alpha value of 0.2. We were able to use the complete AVANT dataset for training giving the utilization of GSE39582 as independent validation dataset. For validation of the signature in an independent dataset (GSE39582), we used only the signs of the fitted coefficients, not the magnitudes, to calculate an unweighted signature score per patient; this was intended to minimize the impact of gene expression platform differences between AVANT and GSE39582. That said, using the fitted coefficients from the model to compute a weighted signature score per patient in GSE39582 yielded qualitatively similar results.

### Pathway and published signature analysis

The four gene clusters within the AVANT signature were determined by correlation and hierarchical clustering analysis. Pathway signatures were obtained from literature: the T-effector signature [31], the stromal gene sets Fibroblast TGFb Response Signature (F-TBRS) [25] and cancer-associated fibroblasts (CAF) [24], and the 12-gene Oncotype Dx Colon Recurrence Score [32]. Representative markers of natural killer (NK) and plasmacytoid dendritic (pDC) cells were manually curated from literature: NK markers KLRK1, KLRC2/3, KLRD1, NKG7, and FCGR3A (CD16) [33–35], and pDC markers CLEC4C, IL3RA, TCF4 and NRP1 [36, 37]. Pathway signatures were calculated as the average Z-score of all the genes contained in each signature, with the exception of the Oncotype Dx Colon Recurrence Score. An Oncotype Dx score was calculated as 0.1263 x stromal group score (average Z-score of BGN, FAP, INHBA)– 0.3158 x cell cycle group score (average Z-score of MYC, MYBL2, MKI67) + 0.3406 x GADD45B, as previously described [32]. In the case of the AVANT cohort, signatures were first restricted to genes on the Nanostring platform before calculating a signature score (e.g. 4/7 cancer-related Oncotype Dx genes, NK marker KLRK1, and pDC markers TCF4 and NRP1). In the context of GZMB, the T-effector signature was modified by removing GZMB and GZMA from the signature which we refer to as “modified T-effector” signature.

### Statistical analysis

Survival analysis with Cox models for genes and pathway signatures were performed in R using the survival package with either continuous values, binarized at median (high vs low expression) or cut into quartiles. Kaplan-Meier curves were generated using the survminer package in R. To test the additive effect of GZMB expression to the gene subsets of the AVANT signature or the additive effect of one signature to another signature, likelihood ratio test p values were calculated using ANOVA on nested models. Multivariate analysis in AVANT was performed by adding the clinical covariates age, levels of Cancer Embryonic Antigen (CEA) in the blood, ECOG status, sex, and a combination of AJCC tumor status (II and III) and lymph node status (N1 and N2), referred to as strata. These covariates were individually found to be prognostic in both OS and DFS. Multivariate analysis in GSE39582 was performed by adding the clinical covariates age, sex, tumor stage (0–4), and lymph node status. These covariates were individually found to be prognostic in both OS and RFS. Note that in GSE39582, we did not have data for ECOG status and CEA blood levels. In GSE39582, the type of adjuvant therapy as well as stage of metastasis were also prognostic clinical covariates. Including them into the multivariate analysis for the AVANT signature yielded similar results as above, hence were excluded from the model for consistency with the covariates in the AVANT dataset. Pearson correlation plots were generated using the corrplot R package. Pairwise T-test was used to compute nominal p-values displayed in the insert tables for boxplots. Chi-square test was used to compute the enrichment of clinical characteristics in CMS subtypes. Most plots were generated using the ggplot2 R package.

### CyTOF data generation and analysis

Disaggregated CRC tumor samples (stage II or III, pre-treated) were procured from an external vendor (Conversant Bio) and analyzed by mass cytometry as previously described [38]. In short, CRC single-cell suspensions were incubated with a cisplatin-based viability dye (Fluidigm), and Human Trustain FcX^™^ block (Biolegend), prior to staining with a 37-parameter isotope conjugated panel of mABs (see S4 Table for clones and vendors). Initial staining of cell surface markers was conducted on live cells and intracellular targets were probed following fixation and permeabilization with the Human Foxp3 Staining Buffer Set (eBioscience). Following intracellular staining cells were fixed with 1.6% paraformaldehyde (Electron Microscopy Sciences) and treated with Cell-ID^™^ Intercalator (Fluidigm). Cells were resuspended in water containing EQ^™^ Calibration Beads prior to acquisition on a Helios upgraded CyTOF 2 (Fluidigm). Signal normalization was conducted as previously described [39].

For each sample, CD45+ viable singlet cells were exported into new.FCS files using FlowJo software. Using 37 immune markers (excluding pan-markers like EPCAM and CD45; markers with broad signal like CD66 and Foxp3) and singlet viable CD45+ populations across the 12 CRC patients, the tSNE dimensionality reduction algorithm (adjClust package, from https://bitbucket.org/cbolen1/adjclust) was used to obtain a tSNE map. Density-based spatial clustering of applications with noise (DBSCAN) via the DBSCAN package in R was applied to the 37 immune markers supplemented with the two axes of the tSNE map, the latter to allow mapping of the DBSCAN clusters onto the tSNE map. Cluster information together with literature-derived knowledge of cell type markers was used to annotate the clusters in the tSNE space. The average of each protein marker was calculated per immune cell type for correlation analysis and heatmaps. In fluorescence-based cytometry experiments, fresh healthy human PBMCs were procured from the Genentech Blood Donors program and processed with a similar protocol as that used for mass cytometry with the exception that fluorophore conjugated mAbs were used and data were acquired on a Canto II instrument.

## Results

### Patient characteristics and tumor profiling

We explored a collection of 1062 FFPE derived patient archival tumors from AVANT, a prospective three-arm randomized phase 3 trial (S2 Fig). The AVANT trial was designed to compare bevacizumab plus oxaliplatin in combination with either FOLFOX4 or capecitabine (XELOX) vs. FOLFOX4 alone, with DFS as a primary endpoint in histologically confirmed stage III or high-risk stage II colon carcinoma [7]. Clinical characteristics in the biomarker evaluable population (BEP) were similar to those in the intent-to-treat population (S5 Table). Since the AVANT trial did not show a significant DFS difference among the arms after a minimum of 3 years follow-up (S1 Fig), we combined all arms in the BEP population for the purpose of identifying a prognostic gene signature in stage II and stage III CRC (S2 Fig). We carried out transcriptional profiling of the FFPE tissues using a customized, CRC-focused NanoString panel (S2 and S3 Tables). The AVANT high risk stage II/III population was comparable to other early stage populations such as GSE39582 [17] and TCGA [16] in terms of prevalence of the four CMS subtypes [12], the enrichment of microsatellite instability high (MSI-H) and BRAF mutations in the CMS1 group, and the enrichment of KRAS mutations in CMS3 (S3 and S4 Figs, S1 Table).

### Identification and performance of a *de novo* prognostic signature for adjuvant CRC

**Fig 1:**
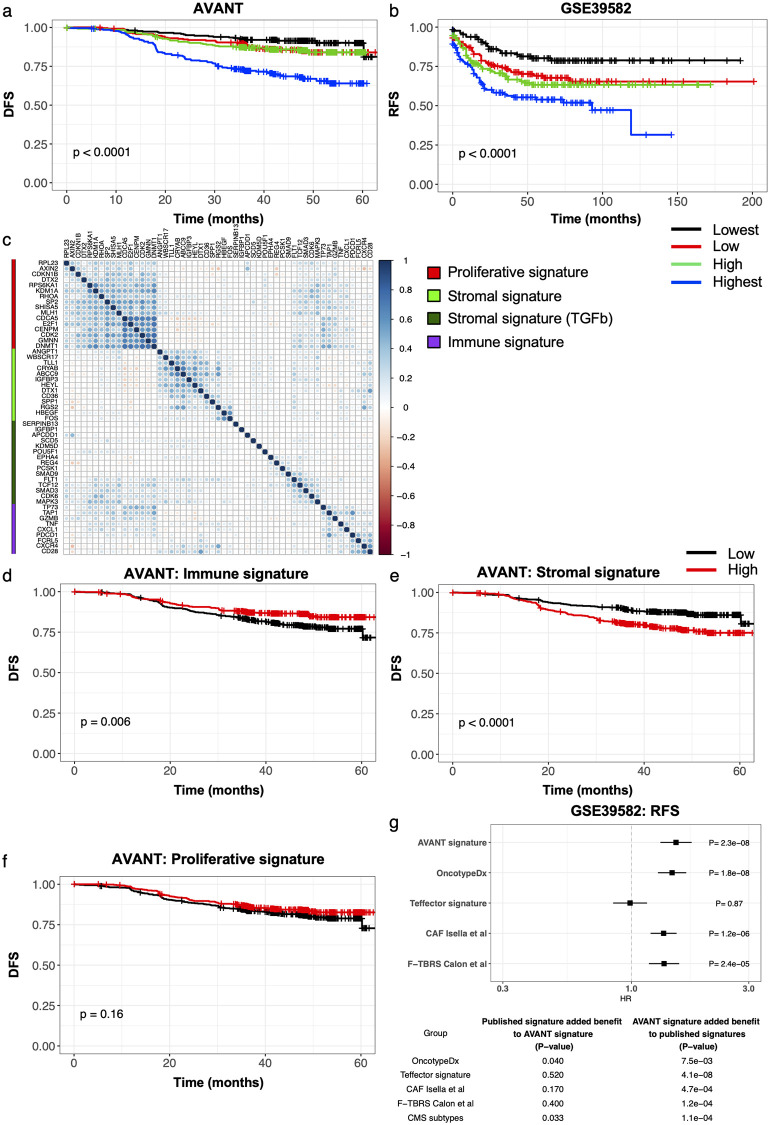
*De novo* prognostic signature for adjuvant CRC. (a) Kaplan-Meier curves for the AVANT signature in the AVANT BEP dataset for DFS. The p-value corresponds to a log-rank test. (b) Kaplan-Meier curves for the AVANT signature in an independent validation cohort GSE39582 for RFS. (c) Correlation plot for the genes in the AVANT signature. Blue denotes positive and red denotes negative Pearson correlation. Color intensity denotes the strength of the correlation. Signature genes were assigned to one of four clusters. Red denotes proliferative genes, light and dark green denote stromal genes, and purple denotes immune genes. (d-f) Kaplan-Meier curves for the prognosis of (d) the immune genes, (e) stromal genes, and (f) proliferative genes of the AVANT signature divided at the median in the AVANT BEP dataset. (g) RFS forest plot for validation dataset GSE39582 shows hazard ratios and associated p-values for each individual signature. In the table below for GSE39582, column (1) assesses significance of added prognostic value (if any) provided by each published signature when added to the AVANT signature; column (2) assesses significance of added prognostic value provided by the AVANT signature when added to each of the individual published signatures.

To interrogate the biological processes associated with survival in adjuvant CRC, we applied an elastic net Cox penalized regression model to the AVANT BEP expression data using OS as the outcome (see Materials and methods and S5 Fig). We identified a highly prognostic *de novo* signature, referred to as the AVANT signature (S6 Table), where high expression of the signature genes was associated with poor prognosis in AVANT for both OS, as expected, and DFS (Fig 1a). This signature showed comparable association with OS and DFS in the three treatment arms of the AVANT trial (S6 Fig; ANOVA signature + treatment arm vs. signature alone, p = 0.25 for OS and p = 0.24 for DFS), confirming the prognostic nature of the signature in the context of standard adjuvant chemotherapy with or without bevacizumab. The signature’s ability to identify a high-risk adjuvant CRC population was validated for recurrence-free survival (RFS) in the independent GSE39582 cohort of stage I-IV colon cancer (Fig 1b, S7 Fig). Importantly, this real-world cohort was not selected for high-risk CRC patients and rather captured patients with stage 0–4 colon cancer. While TNM staging was prognostic in the GSE39582 cohort (HR 2.73, p < 2e-16), the AVANT signature remained prognostic after accounting for stage (ANOVA, p = 7.6e-5), suggestive of the signature’s ability to broadly capture high risk of recurrence across all stages of CRC.

Gene clustering revealed four clusters of genes in the signature that, based on manual curation of the genes per cluster, meaningfully capture three primary biological functions: stromal, proliferative, and immune signaling (Fig 1c, S8 Fig). Although our elastic net approach integrated multiple biological functions into a single signature, we were also interested in the clusters’ individual prognostic contributions. High expression of stromal genes (combining the two stromal clusters) was correlated with poor prognosis while high expression of immune genes was correlated with good prognosis (Fig 1d and 1e). Expression of the proliferative genes was not significantly associated with DFS on its own (Fig 1f). The immune and both sets of stromal genes displayed high expression in the lowest and highest quartile of the AVANT signature respectively, thus further delineating the individual prognostic contributions to the AVANT signature (S8 Fig, Fig 1d–1f). Importantly, the association of immune gene expression with DFS was not only observed in MSI-H patients (HR = 0.31, p = 0.024), which more highly express the immune genes (S9 Fig), but also in MSS patients, though to a lesser extent (HR = 0.74, p = 0.053). In the independent GSE39582 dataset, the stromal and proliferative gene sets were prognostic of RFS, while the immune gene set was not, possibly due to clinical and/or biological inter-cohort variability including tumor stage (S10 Fig).

The AVANT signature was significantly more effective at predicting survival, and thus at identifying a high-risk subpopulation, than other prognostic signatures (Oncotype Dx, T-effector, CAF, F-TBRS) [13, 24, 25] and the CMS subtypes, not only in AVANT as expected (S11 Fig), but more importantly in GSE39582, an independent dataset in which patients were not selected to be high-risk (Fig 1g, S3 Fig). The AVANT signature conferred significant additional prognostic value when combined with each of the previously published prognostic signatures we considered (Fig 1g, bottom table). In contrast, although the oncotype Dx score and CMS subtypes captured recurrence signal beyond what was captured by the AVANT signature, it was still a less significant improvement in RFS prediction compared to adding the AVANT signature to either oncotype Dx score or CMS subtype in both the GSE39582 and AVANT datasets (Fig 1g and S11 Fig). None of the other signatures captured significant additional recurrence signal when combined with the AVANT signature. This suggests that the AVANT signature incorporates the primary drivers of prognosis, and that other signatures offer no or limited additional prognostic value. In addition, multivariate analysis of the AVANT signature incorporating clinical covariates that are prognostic in CRC (listed in Materials and methods) still showed prognostic value of the signature, in AVANT as well as in the independent GSE39582 dataset (S12 Fig).

### GZMB as a highly prognostic, T cell independent biomarker in CRC

**Fig 2:**
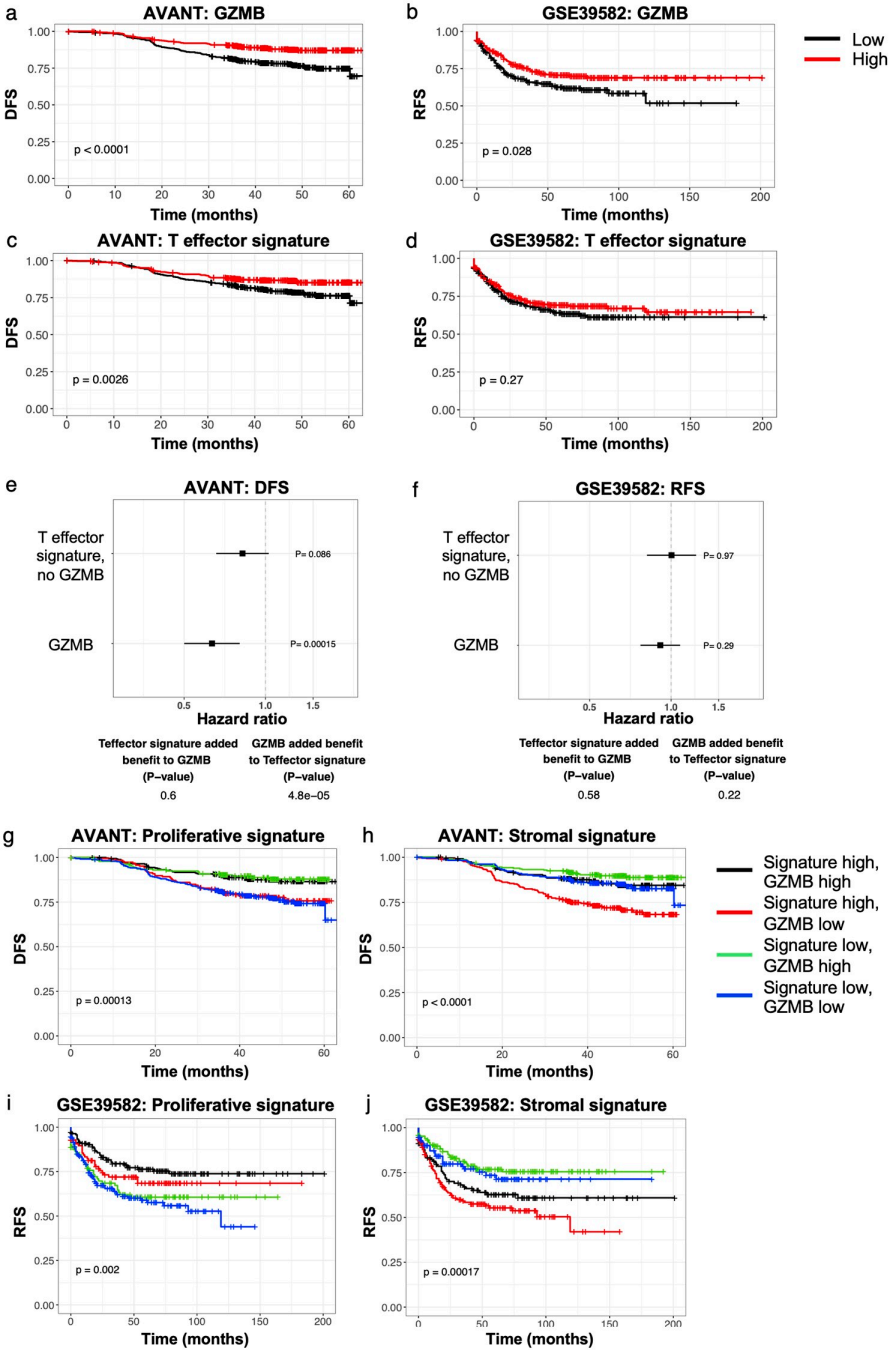
GZMB is a prognostic, T cell independent biomarker in CRC. (a-b) Kaplan-Meier curves for GZMB expression stratified by median in (a) the AVANT BEP dataset for DFS and (b) GSE39582 for RFS. (c-d) Kaplan-Meier curves for the T-effector signature stratified by median expression in (c) the AVANT BEP dataset and (d) GSE39582. (e-f) Forest plots show hazard ratios and associated p-values for the T-effector signature without GZMB and for GZMB expression in (e) the AVANT BEP dataset and (f) GSE39582. The tables below indicate the significance of added prognostic value (if any) provided by the T-effector signature when added to GZMB (first column) and vice versa (second column). Note that continuous GZMB expression in GSE39582 is not prognostic, contrary to high vs. low GZMB expression in panel (b). (g-j) Kaplan-Meier curves showing the relationship between GZMB and (g) the proliferative genes or (h) the stromal genes of the AVANT signature in the AVANT BEP dataset; and between GZMB and (i) the proliferative genes or (j) the stromal genes of the AVANT signature in GSE39582. P-values correspond to a log-rank test.

There is preceding evidence for a role of CD8+ T cells in early disease prognosis in CRC [40–43]. Interestingly, GZMB, a key marker of T-effector cells, is part of our AVANT signature (Fig 1c). Hence we explored the prognostic relevance of singleton GZMB expression versus a previously defined CD8+ T-effector signature [31] in the AVANT and GSE39582 studies. GZMB alone had a significant relationship to prognosis: high expression of GZMB was associated with good prognosis in both the AVANT and GSE39582 datasets (Fig 2a and 2b). In contrast, the T-effector signature as a whole (with GZMB included) was less prognostic in both datasets (Fig 2c and 2d), and the T-effector signature without GZMB added no statistical prognostic value beyond that provided by GZMB alone (Fig 2e and 2f). These observations suggest that GZMB has a distinct prognostic role in CRC that is not captured by other T-effector genes.

We therefore investigated the prognostic value of GZMB in the context of the stromal and proliferative biological functions captured by the AVANT signature. Concurrent high expression of GZMB and the proliferative gene set was associated with the best prognosis in AVANT (Fig 2g). However, this prognostic benefit was lost in patients with a high proliferative gene set but low GZMB expression (Fig 2g). Similarly, high expression of the stromal gene set with low GZMB had the poorest prognosis in AVANT (Fig 2h). Yet, tumors with concurrent high stromal and high GZMB expression were associated with better outcome despite the stromal gene set on its own being associated with poor survival (Fig 2h). The same was true for the prognostic impact of GZMB expression among tumors with a low stromal score. GZMB significantly added prognostic value to the proliferative and stromal gene sets in the AVANT dataset (Fig 2g and 2h; likelihood ratio tests for improvement of fit when adding GZMB expression to the stromal set, p = 4.1e-5, or the proliferative set, p = 1.4e-5), and did not capture significant additional recurrence signal in GSE39582 (Fig 2i and 2j; p = 0.11 and 0.20, resp.). These results indicate that in the AVANT dataset enriched for high-risk patients, high expression of GZMB by itself is consistently associated with favorable outcomes, independent of the proliferative and stromal context.

### Immune cells as a CMS1 and not CMS2-specific source of GZMB

**Fig 3:**
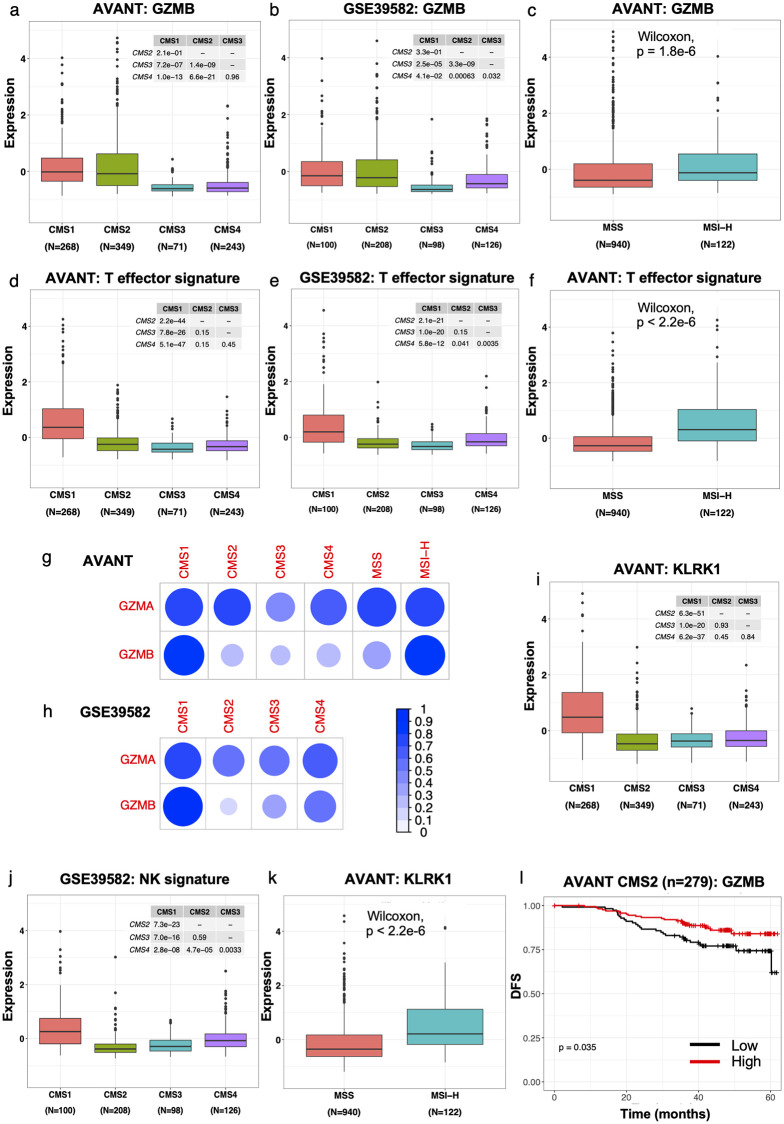
Immune cells are the source of GZMB in CMS1 CRC. (a-b) GZMB expression by CMS subtype in (a) the AVANT BEP dataset and (b) GSE39582. (c) GZMB expression by MSI status in the AVANT BEP dataset. (d-e) Expression of the T-effector signature by CMS subtype in (d) the AVANT BEP dataset and (e) GSE39582. (f) Expression of the T-effector signature by MSI status in the AVANT BEP dataset. (g) Pearson correlation between the modified T-effector signature (i.e. without GZMA/B) and GZMA or GZMB expression in AVANT patients, by CMS subtype or MSI status. (h) Pearson correlation between the modified T-effector signature and GZMA or GZMB expression in GSE39582 patients, by CMS subtype (MSI status was not available). (i) Expression of the NK cell marker KLRK1 by CMS subtype in the AVANT BEP dataset. (j) Expression of the NK signature by CMS subtype in the GSE39582 dataset. (k) Expression of the NK cell marker KLRK1 by MSI status in the AVANT BEP dataset. (l) Kaplan-Meier curve for GZMB expression stratified by median in 279 CMS2 tumors with low T-effector score from the AVANT BEP dataset for DFS. A T-effector score, excluding GZMB, below the mean score in the full cohort is considered low. The p-value corresponds to a log-rank test.

Given the significant impact of this single gene on clinical outcome beyond the prognostic role of other genes associated with CD8+ T cells, we investigated the link between sources of GZMB expression and prognosis in CRC. GZMB was expressed in CMS1 and CMS2 patients in both the AVANT and GSE39582 dataset (Fig 3a and 3b) and was more highly expressed in MSI-H tumors (Fig 3c). Both T-effector and NK cells are established sources of GZMB [44–47]. However, the T-effector signature, including GZMB, was only highly expressed in CMS1 and MSI-H patients (Fig 3d–3f), not in CMS2. T-effector gene GZMA maintained its strong positive correlation with other T-effector genes across CMS subtypes and in both MSI-H and MSS CRC patients (Fig 3g and 3h). GZMB on the other hand was strongly correlated with the T-effector signature only in the CMS1 subtype and in MSI-H CRC patients (Fig 3g and 3h), not in CMS2. Like the T-effector signature, expression of the NK signature (see Materials and methods) was also restricted to primarily CMS1 and MSI-H tumors and showed lowest median expression in CMS2 in both the AVANT and GSE39582 dataset (Fig 3i–3k). These data suggest that the source of GZMB in CRC CMS2 is neither CD8+ T cells nor NK cells. Intriguingly, the CMS2-specific source of GZMB expression has clinical implications: GZMB expression is prognostic in CMS2 tumors with a low T-effector signature, in both AVANT and GSE39582 (Fig 3l and S13 Fig). Taken together, these data suggest that, while CD8+ T and NK cells are a major source of GZMB in CMS1 and MSI-H patients, other cell types may be the source of GZMB—and thus of the prognostic relevance of GZMB expression—in CMS2 patients.

**Fig 4:**
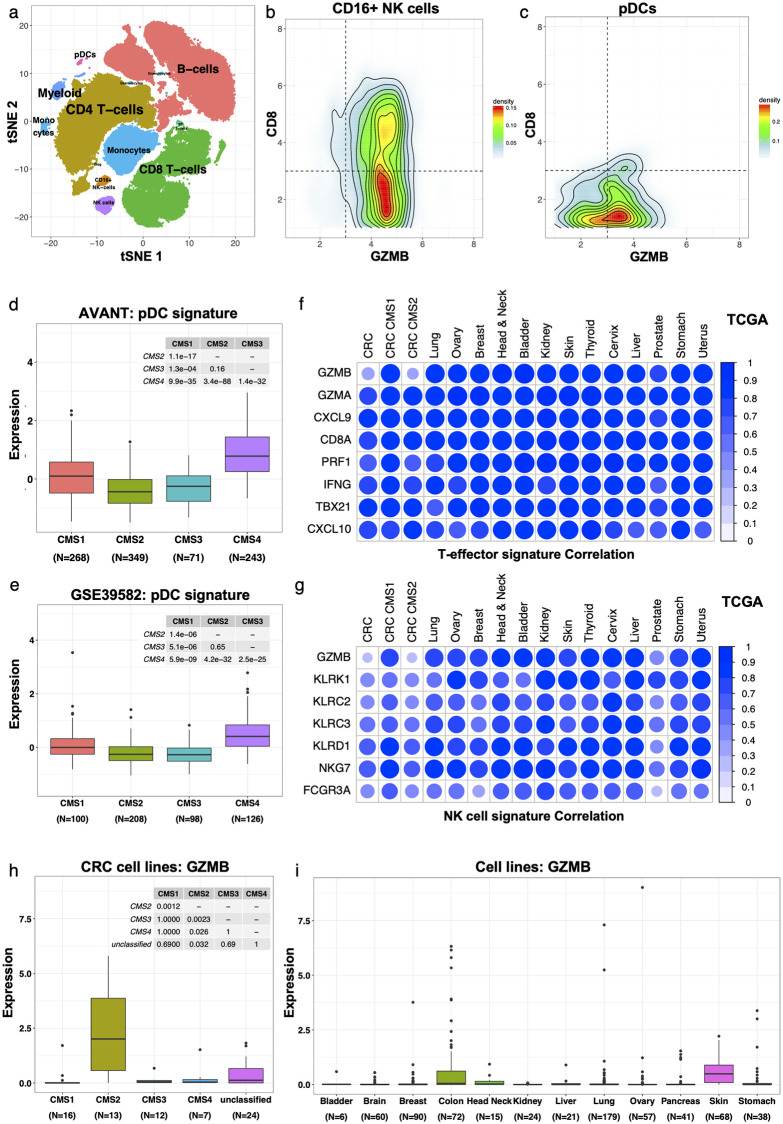
CMS2 CRC expresses endogenous GZMB. (a) tSNE map of the CD45+ immune cell populations in 12 procured CRC samples. Clusters representing different cell types are denoted by distinct colors, based on marker expression (see Materials and methods and S14a Fig). Outliers detected by density-based clustering were excluded. (b-c) CD8 versus GZMB expression in (b) CD16+ NK cells and (c) pDCs. (d-e) Expression of the pDC signature by CMS subtype in the (d) AVANT and (e) GSE39582 dataset. (f) Pearson correlation between expression of single genes from the T-effector signature and the average expression of the other T-effector genes without the gene in question, by cancer type and, for colon, by CMS subtype, in TCGA data. (g) Pearson correlation between expression of single genes from the NK signature and the average expression of the other NK genes without the gene in question, by cancer type and, for colon, by CMS subtype, in TCGA data. (h) GZMB expression (log2 nRPKM+1) by CMS subtype in CRC cell lines. (i) GZMB expression (log2 nRPKM+1) by cancer type in a cohort of 671 cell lines covering 12 cancer types.

To identify novel immune cell types that express GZMB, we conducted mass cytometry analysis in 12 resected stage II or III CRC tumors using a comprehensive panel of 37 lineage and functional markers of immune populations (S4 Table). We applied t-distributed stochastic neighbor embedding (tSNE) analysis on the CD45+ viable singlet gated immune cell populations from all 12 CRC patients combined, followed by unsupervised density-based clustering (Fig 4a, Materials and methods). B cells and CD4+ T cells were most prevalent in this cohort of 12 CRC patients, followed by CD8+ T cells and monocytes (S14a and S14b Fig). Novel cell types with average GZMB expression exceeding GZMA levels were CD16+ NK cells and plasmacytoid dendritic cells (pDCs) (S14a Fig), and these cells had primarily low CD8 expression (Fig 4b and 4c, S14c Fig). These data suggest that infiltration of CD16+ NK cells and pDCs may contribute to total GZMB expression in the 12 CRC patients, and this is consistent with an earlier observation of GZMB expression by the above cell types in the absence of detectable Perforin [48, 49]. However, only 1% of the pooled immune cells of the 12 CRC patients are CD16+ NK cells or pDCs. In addition to the low prevalence, both the NK signature (including CD16, aka FCGR3A) and a transcriptional signature of pDCs (see Materials and methods) are low expressed in CMS2 tumors in the AVANT and GSE39582 datasets (Fig 3i and 3j for NK, Fig 4d and 4e for pDC). Together these results suggest that T-effector cells, NK cells and pDCs are a source of GZMB expression in CMS1 tumors, but not in CMS2 tumors.

To address whether this observation is specific to CRC, we investigated the correlation of GZMB expression with the T-effector, NK and pDC cell signatures in the TCGA dataset covering 14 cancer types (Fig 4f and 4g and S14d Fig). The T-effector and NK signature genes were highly correlated in expression in all cancer types considered (Fig 4f and 4g). GZMB expression on the other hand correlated well with these signatures in all cancers except for CRC CMS2 (Fig 4f and 4g), confirming this phenomenon to be CRC CMS2-specific. Correlation among pDC signature genes was in general poor across cancer types (S14d Fig).

### Unique characterization of CMS2 CRC by endogenous GZMB expression

Given these findings and the extensively profiled immune cell populations in Fig 4a, we hypothesized that the source of GZMB expression in CMS2 tumors may be the tumor cells themselves. Indeed, in a cohort of 72 colon cancer cell lines, GZMB was primarily expressed in CMS2 cell lines (Fig 4h). Beyond CMS2 CRC, GZMB expression was also observed in melanoma tumor cell lines across a broader cell line panel covering 12 cancer types (Fig 4i). In contrast, other classical markers of T-effector, NK and pDC cells were not expressed in cancer cell lines (S15 Fig), supporting the hypothesis that GZMB is produced by tumor cells in CMS2 CRC tumors and primarily by immune cells in CMS1 CRC tumors. Of note, cytolytic gene perforin (PRF1) was uniquely expressed in colon cancer cell lines, with a trend towards higher expression in CMS2 CRC lines (S15c and S15d Fig).

## Discussion

This retrospective analysis of the phase 3 AVANT trial identified a prognostic signature that is robust, reproducible and outperforms other established prognostic and recurrence prediction signatures in CRC. Pending clinical validation in a prospective study, this prognostic signature has potential clinical utility to guide therapeutic decisions for patients with stage II or III CRC, to replace existing prognostic classification methods, and to complement the existing TNM classification and also the newly adopted CMS tumor classification systems. Specifically, it allowed us to define a patient population in early stage CRC that is most likely to relapse, as well as to identify low-risk patients that are unlikely to benefit from adjuvant chemotherapy after surgery. The AVANT signature further uncovered three key biological functions associated with disease progression in high-risk adjuvant CRC patients: (1) proliferative, (2) stromal and (3) immune. Each of these has previously been shown to be relevant for prognosis in early stage CRC in isolation [3, 13, 22, 24, 25], but here they are described together for the first time. This finding indicates that both a tumor’s underlying biology and that of its microenvironment should be considered when classifying high-risk stage II/III colon cancer.

Despite the broad therapeutic benefit of checkpoint inhibitor (CPI) treatment across a variety of solid tumor indications, including MSI-high CRC [50], these agents have demonstrated little activity in MSS CRC tumors. We therefore wanted to further investigate the cluster of immune-related genes that were prognostic in MSS CRC tumors. As previously shown, Perforin-, Granzyme A (GZMA)- and GZMB-dependent cytolytic function is acquired during differentiation of naive CD8+ T cells into CD8+ T-effector cells in response to antigenic stimulation [51–56]. Importantly, several clinical studies have shown that high levels of baseline T-effector signatures correlate with improved outcome in CPI treated patients [31, 57], thus suggesting that CPI activity requires pre-existent tumor T-effector immunity. However, our analyses show that GZMB alone can have a significant impact on survival and is associated with good prognosis. In fact, the rest of the T-effector genes as a signature are not prognostic without GZMB. These findings are important given the assumption so far that GZMB and the T-effector signature or CD8 T cells are synonymous. We describe herein a novel and unique role for GZMB as a highly prognostic gene in CMS1 and CMS2 CRC, one that extends beyond the well-known prognostic role of CD8+ T-effector cells in CMS1. GZMB added significant prognostic value to the stromal and proliferative subsets of the AVANT signature. This further supports the need for a cohesive signature and not just immune or stromal to fully capture the biology that drives prognosis and disease pathogenesis in CRC.

Next, in our search to identify non-CD8 T immune cell types that express GZMB, we realized upon further analyses that none of the immune cells identified were the source of GZMB expression in CMS2 CRC. The data from the cohort of 72 colon cancer cell lines, confirmed that GZMB is primarily expressed in CMS2 cell lines and that GZMB expression there is rather a tumor-intrinsic property. We hypothesize that as a potent extracellular matrix (ECM) remodeling agent, GZMB efficiently cleaves vitronectin, fibronectin, and laminin [58]. As shown before, GZMB pretreatment of a laminin matrix significantly inhibited cell spreading of colon cancer cell line LIM1215 *in vitro* [58]. Thus, via disruption of integrin-dependent adhesion, GZMB has been shown to inhibit tumor cell spreading, migration, and invasion on ECM, thereby potentially inhibiting tumor growth and invasion. Here we identified CRC CMS2 as one setting where GZMB is expressed by non-cytolytic cells, and this was established in both CRC cell lines and multiple independent cohorts of CRC. GZMB’s putative role in inhibiting tumor growth and invasion is consistent with our observation that endogenous GZMB expression is associated with favorable outcome in adjuvant CRC (Fig 3l, S13 Fig). The fact that we observed GZMB expression exclusively in *in vitro* CRC CMS2 and melanoma tumor cells across a cell line panel covering 12 cancer types (Fig 4i) emphasizes the need for new research on GZMB function in tumors. This data suggests that the established Immunoscore may only capture the proportion of patients whose tumors are driven by CD8+ T-effector biology and that harnessing this alternative GZMB source from tumor cells is worth exploring as a biomarker and to uncover novel biology in CRC.

Key limitations of this study concern the use of the Nanostring platform with available expression for 829 genes. Even though this gene set was customized for colorectal cancer and hence is powered to capture biologies that are relevant to this disease, full-transcriptome profiling with technologies such as RNA-sequencing could have revealed different individual prognostic genes. We also may have used certain prognostic/predictive signatures and tumor classification methods outside the scope of what they were originally designed for. Finally, more research on the specific role of tumor-intrinsic GZMB in the CMS2 subtype is warranted. That may be achieved through among others mechanistic studies in CMS2 CRC cell lines with GZMB knockdown and/or pharmacologic inhibitors of processes such as ECM cleavage.

## Conclusions

While few therapeutics have shown promise in CRC, the data described herein enabled the discovery of new underlying biology in this indication and uncovered a tissue-based signature that may not only guide treatment allocation but also help improve the selection of high-risk resectable CRC patients in adjuvant trials.

## Supporting information

S1 Fig Kaplan-Meier curves for the three treatment arms in the AVANT BEP dataset for (a) OS, and (b) DFS. The p-value corresponds to a log-rank test.
(PDF)

S2 Fig CONSORT diagram for the biomarker analyses in the AVANT trial. (PDF)

S3 Fig (a-c) Percentage of KRAS mutation by CMS subtype in (a) the AVANT BEP dataset, (b) TCGA, and (c) GSE39582. (d-f) Percentage of BRAF mutation by CMS subtype in (d) the AVANT BEP dataset, (e) TCGA, and (f) GSE39582. (g-h) Percentage of MSI-H vs. MSS by CMS subtype in (g) the AVANT BEP dataset and (h) TCGA. MSI status was not available for GSE39582. (i) Percentage of right versus left sidedness of the colon by CMS subtype in the AVANT BEP dataset. Chi-square test p-values are noted below the legend in each panel.
(PDF)

S4 Fig (a) Expression of 132 CMS classifier genes (rows) in 1062 AVANT patients (columns). The predicted CMS subtypes are denoted for each patient on top. The row annotation denotes the subtype in which a gene is uniquely high expressed. We refer to materials and methods for details on the CMS classification strategy. (b) Expression of 132 CMS classifier genes in TCGA CRC patients, with genes shown in the same order as in panel (a). The CMS subtypes as predicted by the Guinney et al random forest algorithm are denoted for each patient on top. (c) Percentage of the CMS subtypes in the AVANT BEP dataset. Patients that lack CMS classifier gene expression for any subtype are labeled as unclassifiable. (d) Percentage of the CMS subtypes in TCGA. (e) Expression of 132 CMS classifier genes in the GSE39582 dataset, with genes shown in the same order as in panel (a). The CMS subtypes as predicted by the Guinney et al random forest algorithm are denoted for each patient on top. (f) Percentage of the CMS subtypes in the GSE39582 dataset.
(PDF)

S5 Fig (a) Cox-based elastic net regression results for the identification of genes prognostic for OS in AVANT, using alpha values ranging from 0.1 to 0.9. Identified prognostic genes per alpha value are denoted by black tiles. As expected for elastic net approaches, lower alpha values usually result in larger numbers of selected genes, i.e., less sparse fitted models. We selected genes at alpha 0.2 for our prognostic signature (see Materials and methods). (b) Cross-validation accuracy results for the selected alpha = 0.2 model.
(PDF)

S6 Fig Kaplan-Meier curves for the AVANT signature in the AVANT BEP dataset split by treatment arm as indicated, for DFS: (a) FOLFOX-4, (b) FOLFOX-4 + bevacizumab, and (c) XELOX + bevacizumab; and for OS: (d) FOLFOX-4, (e) FOLFOX-4 + bevacizumab, and (f) XELOX + bevacizumab. P-values correspond to a log-rank test.
(PDF)

S7 Fig (a) Kaplan-Meier curves for the AVANT signature with use of weighted fitted coefficients in an independent validation cohort GSE39582 for RFS. (b) RFS forest plot for validation dataset GSE39582 shows hazard ratios and associated p-values for each individual signature, with use of weighted fitted coefficients for the AVANT signature as in panel (a). In the table below for GSE39582, column (1) assesses significance of added prognostic value (if any) provided by each published signature when added to the AVANT signature; column (2) assesses significance of added prognostic value provided by the AVANT signature when added to each of the individual published signatures.
(PDF)

S8 Fig (a-d) Average expression of the four gene clusters of the AVANT signature from Fig 1c, by the AVANT signature quartiles in the AVANT BEP dataset. (e-h) Average expression of the four gene clusters of the AVANT signature by CMS subtype in the AVANT BEP dataset. The table insets show p-values computed using the pairwise T-test for each comparison adjusted for multiplicity testing. (i) Expression of the AVANT signature genes (rows) in the AVANT BEP dataset (columns). Red denotes high expression and blue denotes low expression. Patients are annotated by CMS subtype and the quartile of the AVANT signature. Row annotation indicates the respective signatures from Fig 1c.
(PDF)

S9 Fig (a-c) Average expression of the gene clusters of the AVANT signature from Fig 1c by MSI status in the AVANT BEP dataset. The two stromal gene clusters were combined for panel (c). P-values correspond to a T-test. (d) Correlation between the average expression of the gene clusters of the AVANT signature in the AVANT BEP dataset, with the two stromal gene clusters combined. Pearson correlations are denoted, with font size reflecting significance.
(PDF)

S10 Fig (a-c) Kaplan-Meier curves for the gene clusters of the AVANT signature from Fig 1c (combining the two stromal gene clusters) stratified by median, in the GSE39582 dataset for RFS. P-values correspond to a log-rank test.
(PDF)

S11 Fig DFS forest plot shows hazard ratios and associated p-values for each individual signature in the AVANT BEP dataset. The table below indicates the significance of added prognostic value (if any) provided by each published signature when added to the AVANT signature (first column) and vice versa (second column).
(PDF)

S12 Fig Multivariate analysis results for the AVANT signature. Forest plots denote the Cox hazard ratios (HR) and p-values for the different signature quartiles after adjustment for clinical covariates. Each individual clinical covariate in each trial was tested for its effect on prognosis (DFS or RFS) and only prognostic covariates were included in the multivariate analyses. (a) Included covariates in the AVANT BEP dataset were age, sex, level of CEA in blood, ECOG status and AJCC tumor status including lymph node status (i.e. strata). (b) Included covariates in the GSE39582 dataset were age, sex, tumor stage, and lymph node status.
(PDF)

S13 Fig Kaplan-Meier curve for GZMB expression stratified by median in 177 CMS2 tumors with low T-effector score from the GSE39582 dataset for RFS. A T-effector score, excluding GZMB, below the mean score in the full cohort is considered low. The p-value corresponds to a log-rank test.
(PDF)

S14 Fig (a) Average expression of each marker across the detected immune cell clusters from Fig 4a. Shown are row scaled arcsinh transformed intensity values. Red denotes high expression; blue denotes low expression. (b) Percentage of different immune cell populations across the 12 CRC patients. (c) CD8 versus GZMB expression in CD8+ T cells. (d) Pearson correlation between expression of single genes from the pDC signature and the average expression of the other pDC genes without the gene in question, by cancer type and, for colon, by CMS subtype, in TCGA data.
(PDF)

S15 Fig (a-b) Expression (log2 nRPKM+1) of T-effector marker GZMA in (a) CRC cell lines by CMS subtype and (b) a cohort of 671 cell lines covering 12 cancer types. (c-d) Expression (log2 nRPKM+1) of T-effector marker PRF1 in (c) CRC cell lines by CMS subtype and (d) a cohort of 671 cell lines covering 12 cancer types. (e-f) Expression (log2 nRPKM+1) of NK cell marker CD16 (FCGR3A) in (e) CRC cell lines by CMS subtype and (f) a cohort of 671 cell lines covering 12 cancer types. (g-h) Expression (log2 nRPKM+1) of pDC cell marker CLEC4C in (g) CRC cell lines by CMS subtype and (h) a cohort of 671 cell lines covering 12 cancer types.
(PDF)

S1 Table Clinical characteristics of the biomarker evaluable population of 1062 CRC tumors. (XLSX)

S2 Table Overview of the customized NanoString panel with 829 CRC-associated genes, 7 negative controls, and 6 positive controls. (XLSX)

S3 Table Nanostring z-scored expression data for 829 CRC-associated genes in 1062 FFPE derived patient archival tumors from the AVANT trial. (XLSX)

S4 Table Overview of clones and vendors of a 37-parameter isotopic conjugated panel of monoclonal antibodies for lineage and functional markers of immune populations. (PDF)

S5 Table Clinical characteristics in the Intent-to-Treat Population by treatment arm and the biomarker evaluable population. (PDF)

S6 Table Prognostic signature specification including genes, model coefficients, and the biology captured by each signature gene. (XLSX)
