# Supplementary figures and images for: Transcriptomic profiling of adjuvant colorectal cancer identifies three key prognostic biological processes and a disease specific role for granzyme B
Source: PLoS One. 2021 Dec 31;16(12):e0262198. doi: 10.1371/journal.pone.0262198 (PMC8719661; doi:10.1371/journal.pone.0262198)

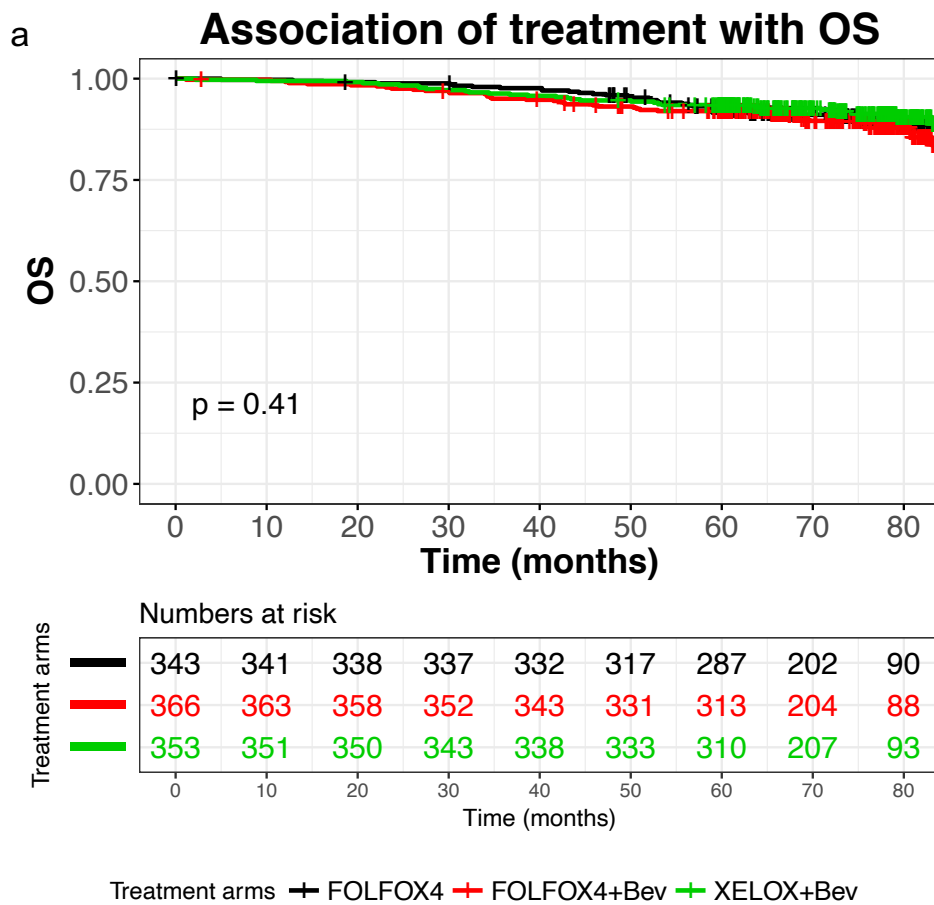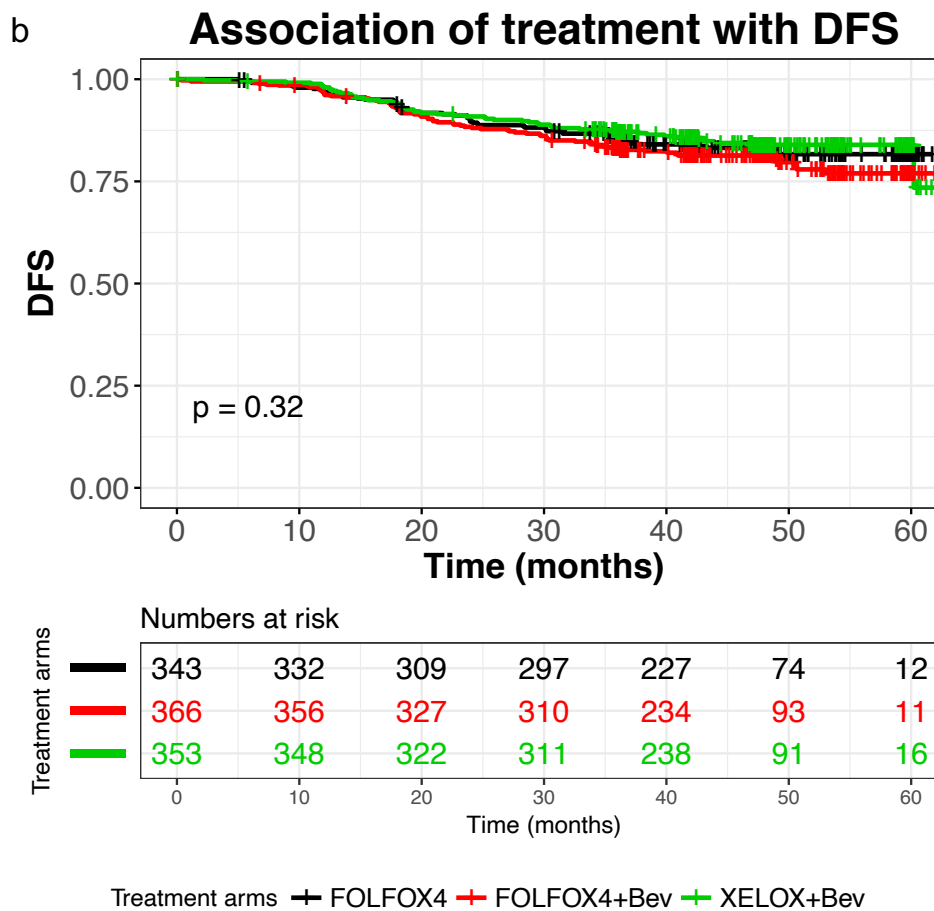

Supplement: S1 Fig — The p-value corresponds to a log-rank test. (PDF) [file pone.0262198.s001.pdf]

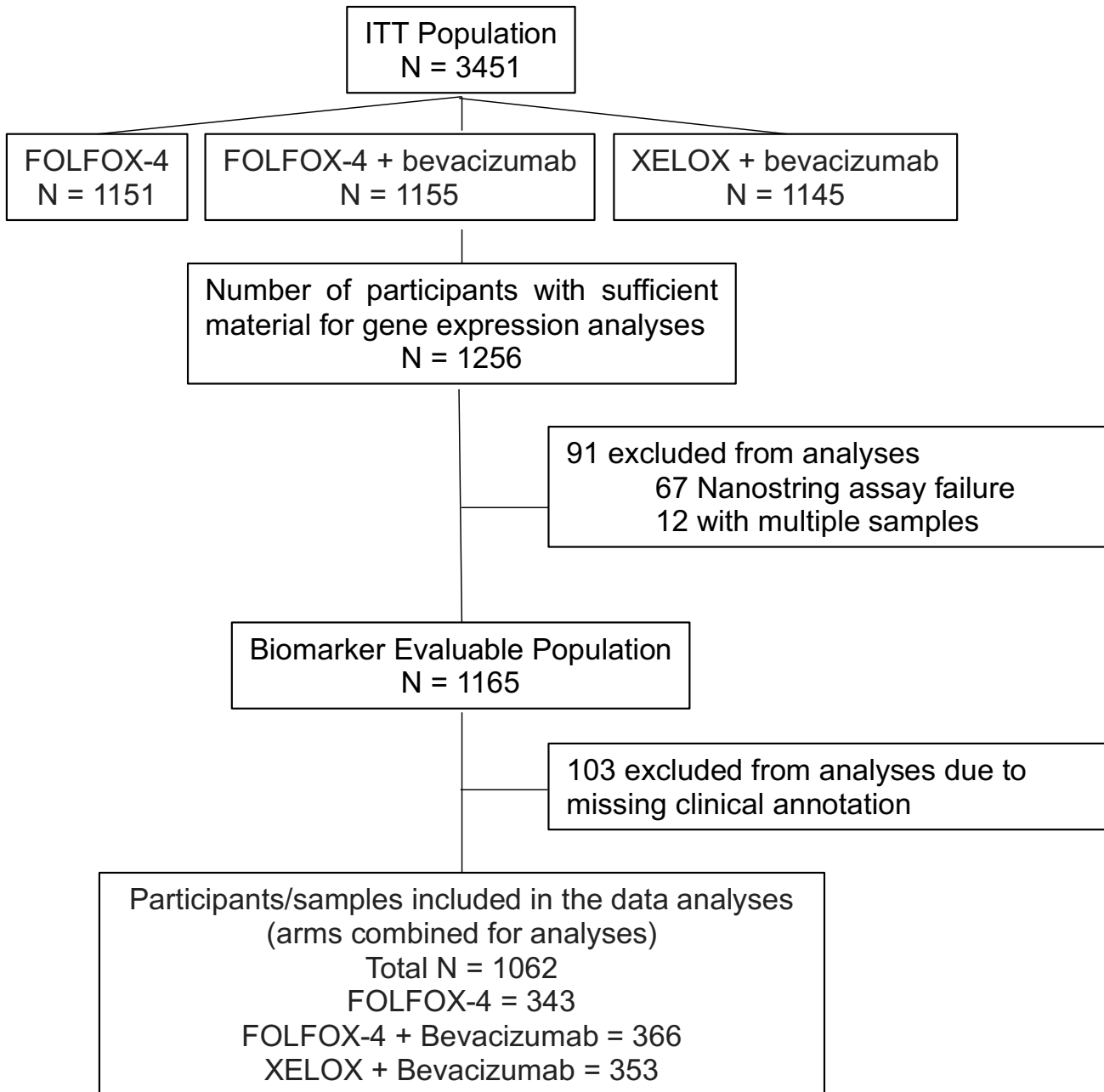

Supplement: S2 Fig — (PDF) [file pone.0262198.s002.pdf]

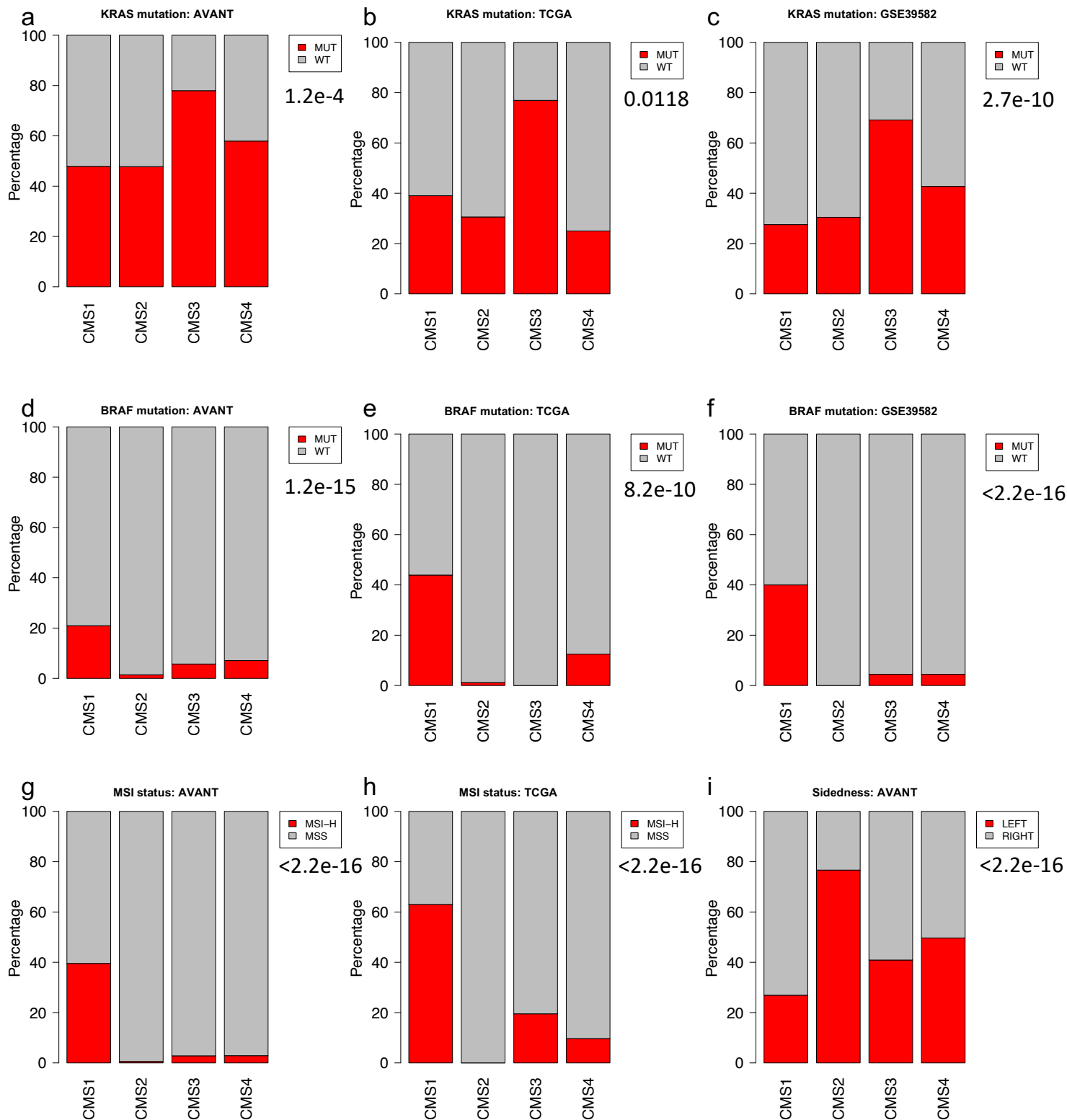

Supplement: S3 Fig — (a-c) Percentage of KRAS mutation by CMS subtype in (a) the AVANT BEP dataset, (b) TCGA, and (c) GSE39582. (d-f) Percentage of BRAF mutation by CMS subtype in (d) the AVANT BEP dataset, (e) TCGA, and (f) GSE39582. (g-h) Percentage of MSI-H vs. MSS by CMS subtype in (g) the AVANT BEP dataset and (h) TCGA. MSI status was not available for GSE39582. (i) Percentage of right versus left sidedness of the colon by CMS subtype in the AVANT BEP dataset. Chi-square test p-values are noted below the legend in each panel. (PDF) [file pone.0262198.s003.pdf]

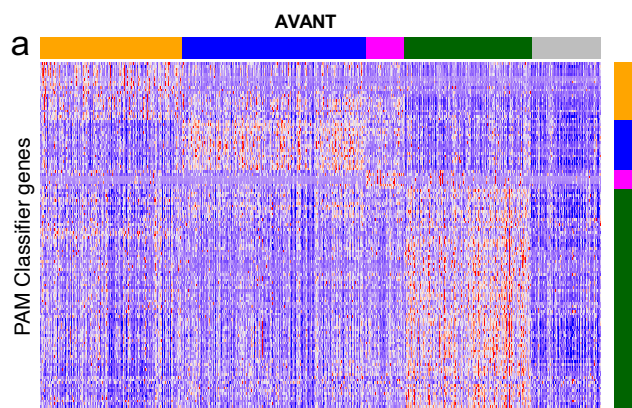

CMS classes: AVANT patients

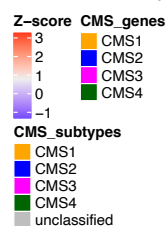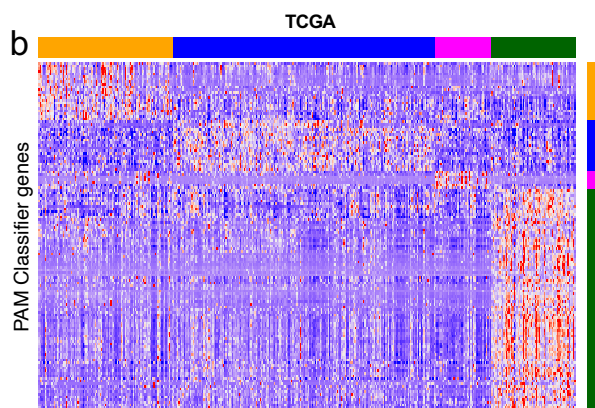

CMS classes: TCGA patients

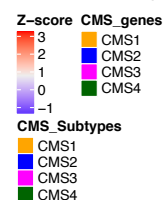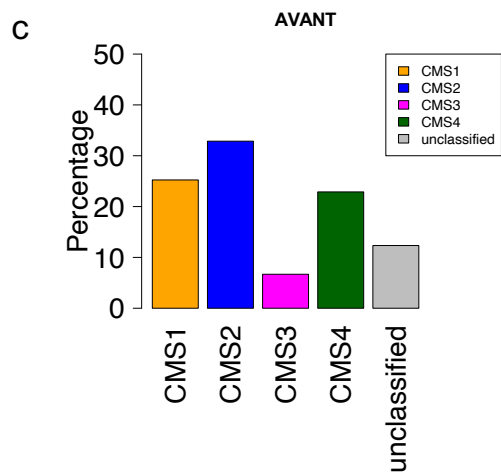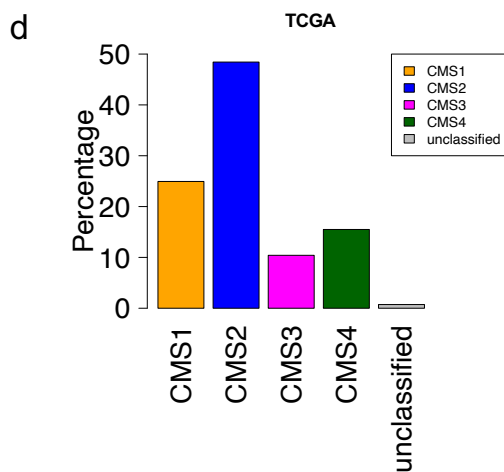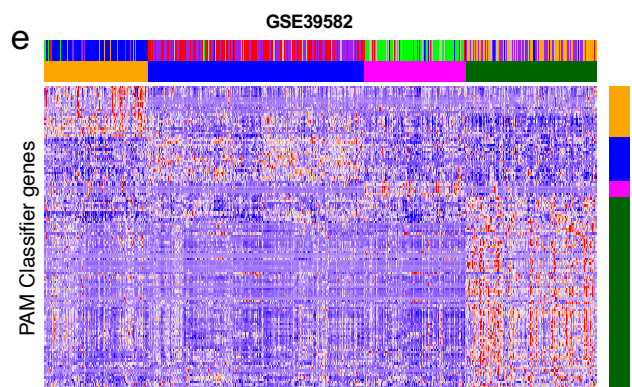

CMS classes: GSE39582 patients

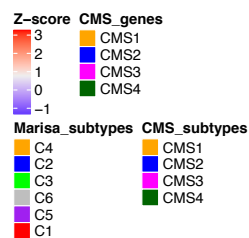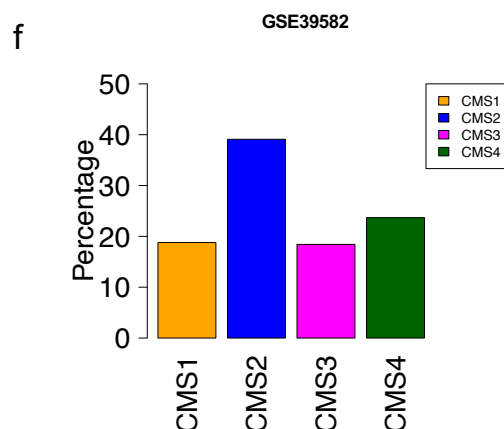

Supplement: S4 Fig — (a) Expression of 132 CMS classifier genes (rows) in 1062 AVANT patients (columns). The predicted CMS subtypes are denoted for each patient on top. The row annotation denotes the subtype in which a gene is uniquely high expressed. We refer to materials and methods for details on the CMS classification strategy. (b) Expression of 132 CMS classifier genes in TCGA CRC patients, with genes shown in the same order as in panel (a). The CMS subtypes as predicted by the Guinney et al random forest algorithm are denoted for each patient on top. (c) Percentage of the CMS subtypes in the AVANT BEP dataset. Patients that lack CMS classifier gene expression for any subtype are labeled as unclassifiable. (d) Percentage of the CMS subtypes in TCGA. (e) Expression of 132 CMS classifier genes in the GSE39582 dataset, with genes shown in the same order as in panel (a). The CMS subtypes as predicted by the Guinney et al random forest algorithm are denoted for each patient on top. (f) Percentage of the CMS subtypes in the GSE39582 dataset. (PDF) [file pone.0262198.s004.pdf]

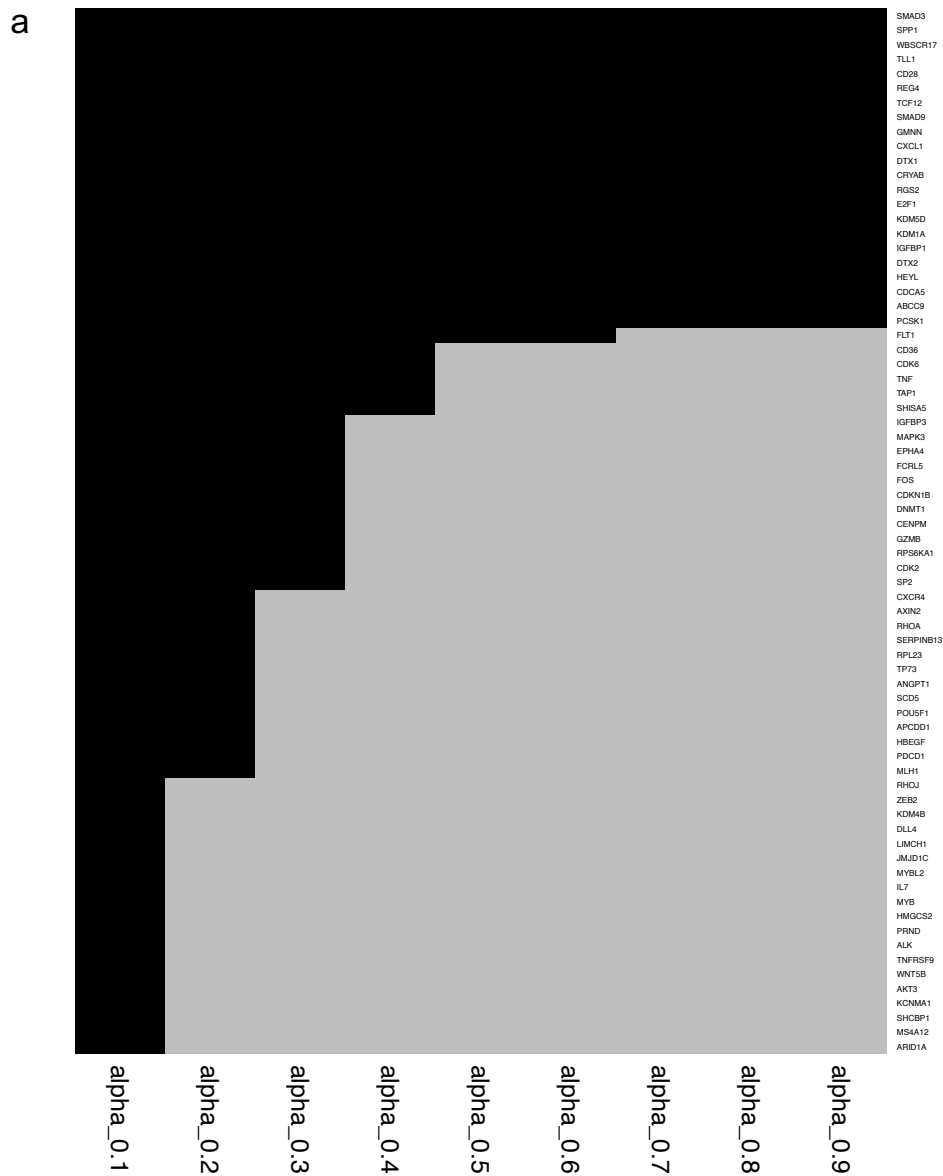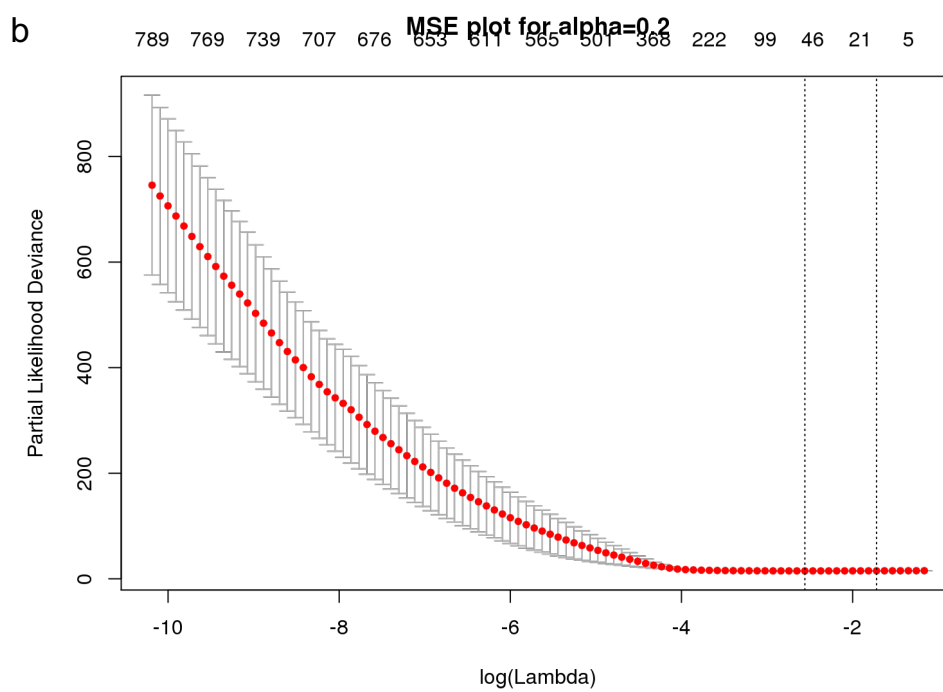

Supplement: S5 Fig — (a) Cox-based elastic net regression results for the identification of genes prognostic for OS in AVANT, using alpha values ranging from 0.1 to 0.9. Identified prognostic genes per alpha value are denoted by black tiles. As expected for elastic net approaches, lower alpha values usually result in larger numbers of selected genes, i.e., less sparse fitted models. We selected genes at alpha 0.2 for our prognostic signature (see Materials and methods). (b) Cross-validation accuracy results for the selected alpha = 0.2 model. (PDF) [file pone.0262198.s005.pdf]

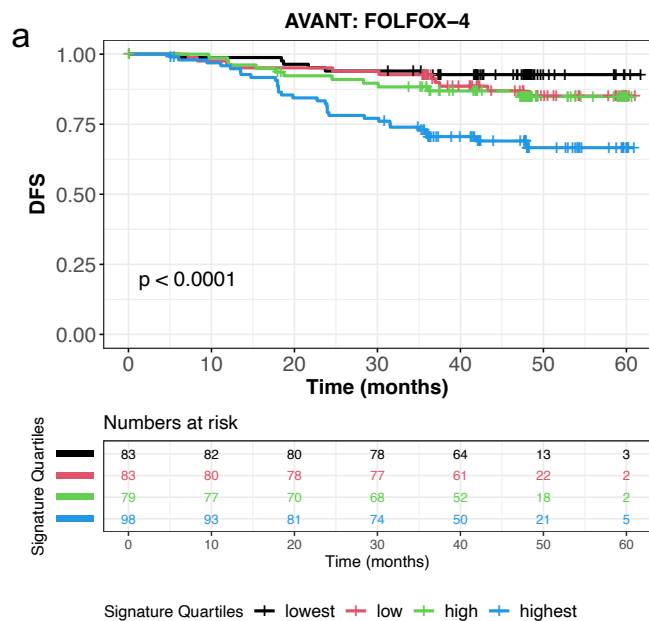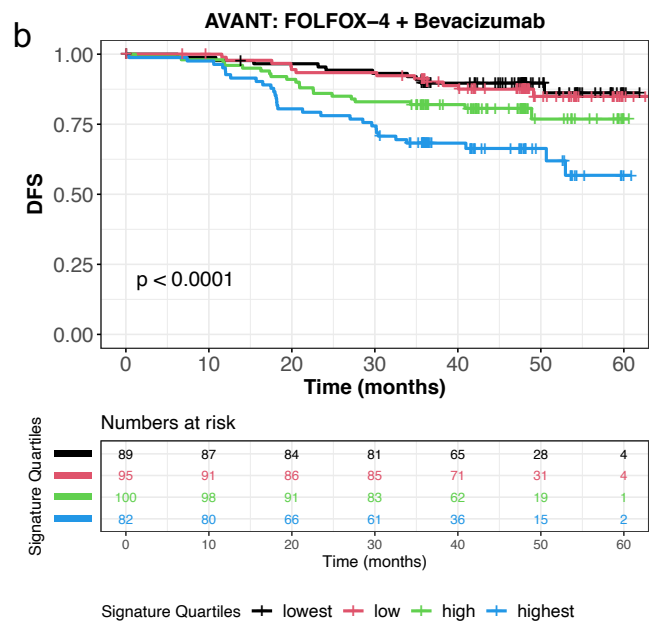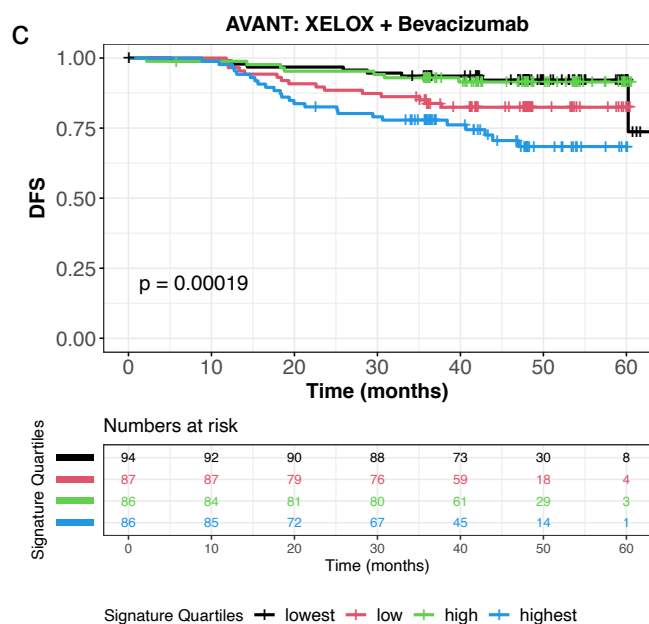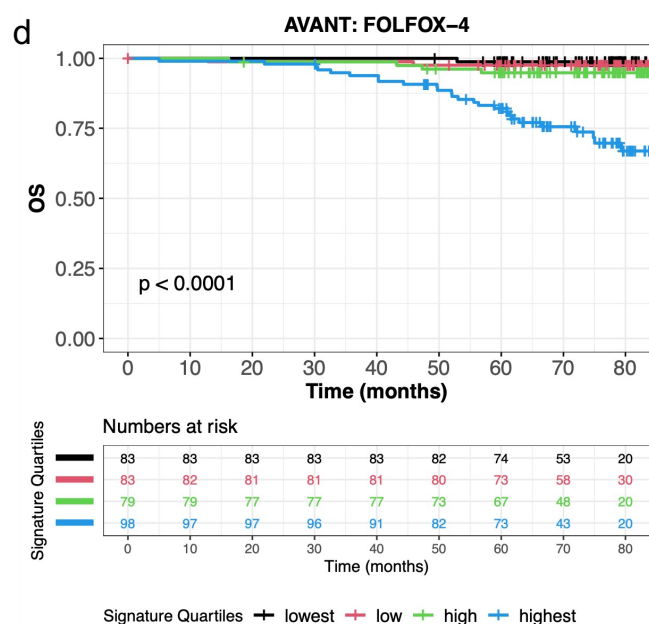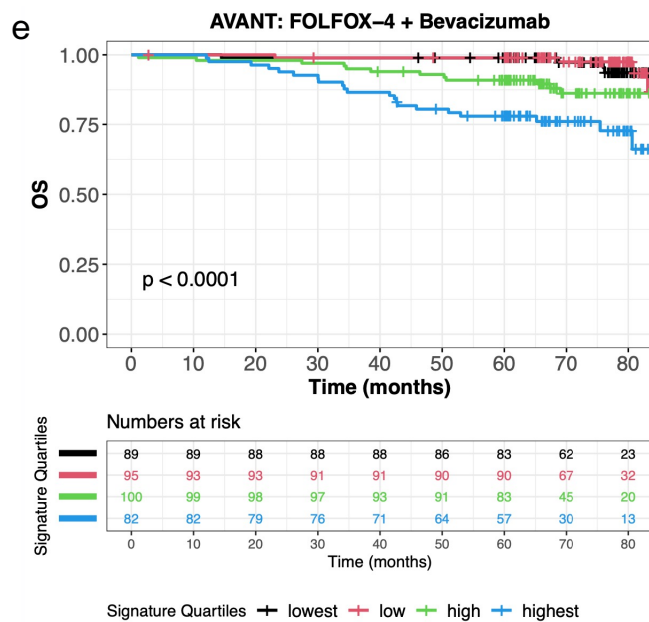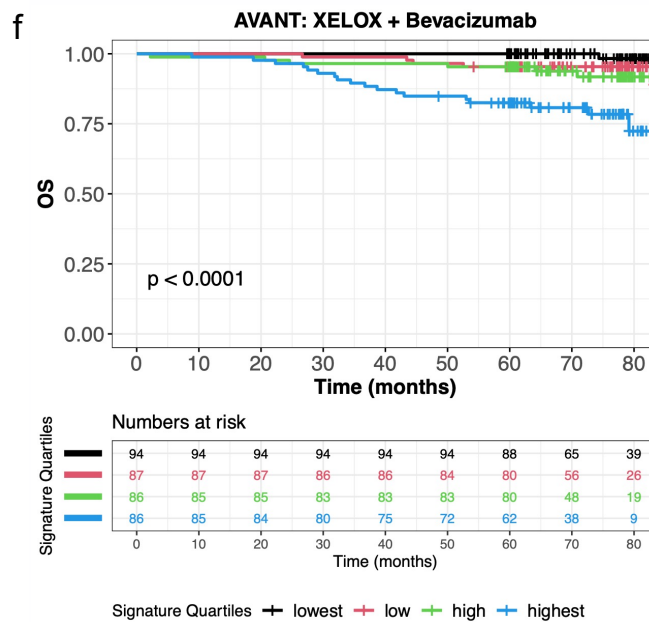

Supplement: S6 Fig — Kaplan-Meier curves for the AVANT signature in the AVANT BEP dataset split by treatment arm as indicated, for DFS: (a) FOLFOX-4, (b) FOLFOX-4 + bevacizumab, and (c) XELOX + bevacizumab; and for OS: (d) FOLFOX-4, (e) FOLFOX-4 + bevacizumab, and (f) XELOX + bevacizumab. P-values correspond to a log-rank test. (PDF) [file pone.0262198.s006.pdf]

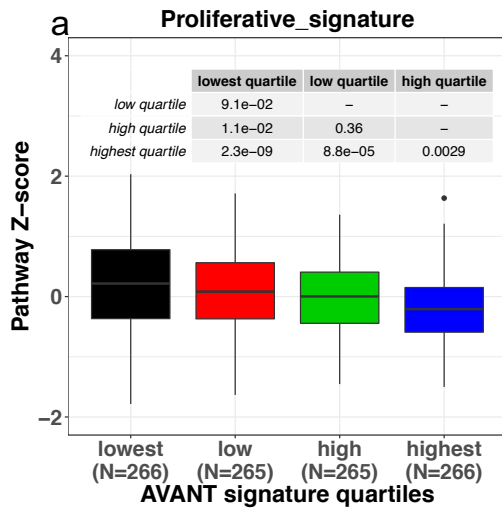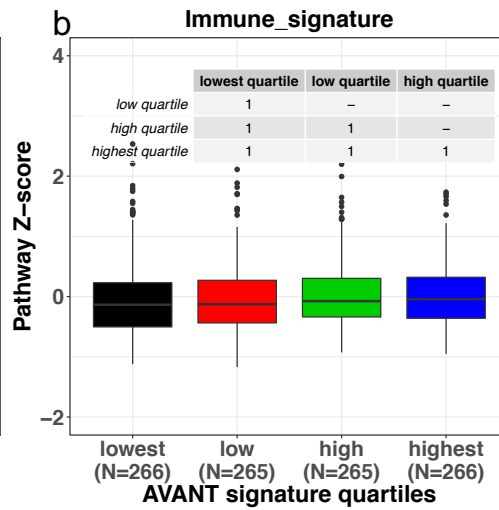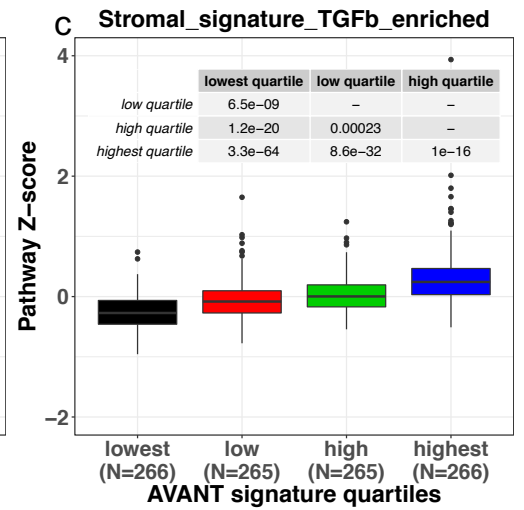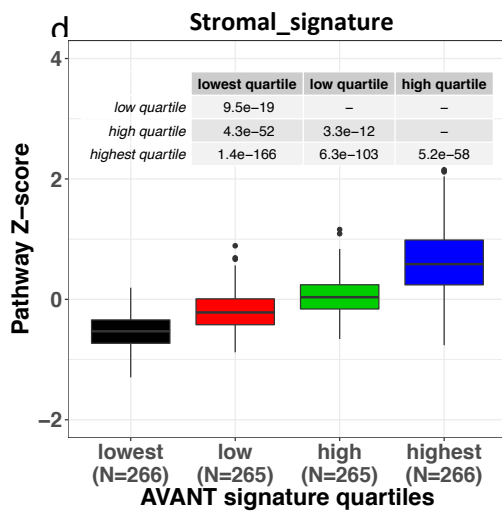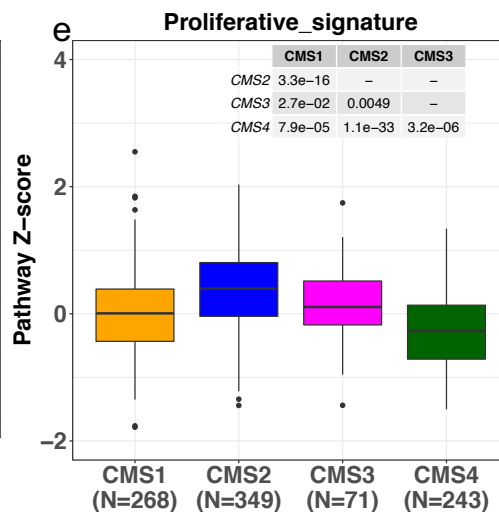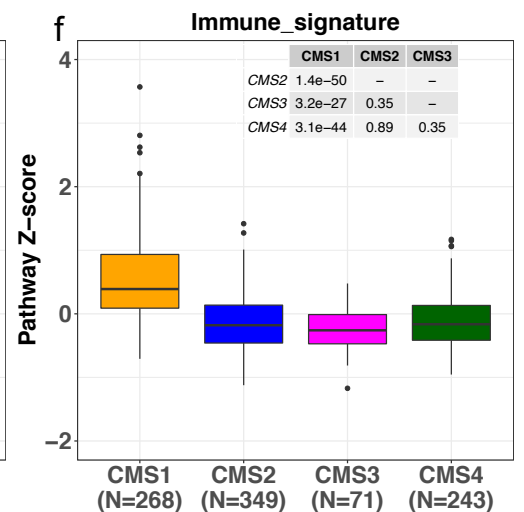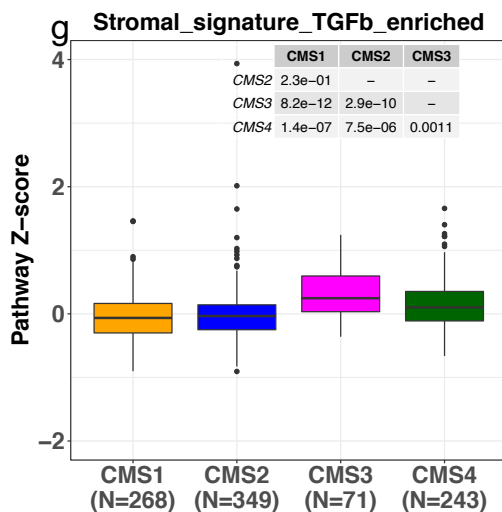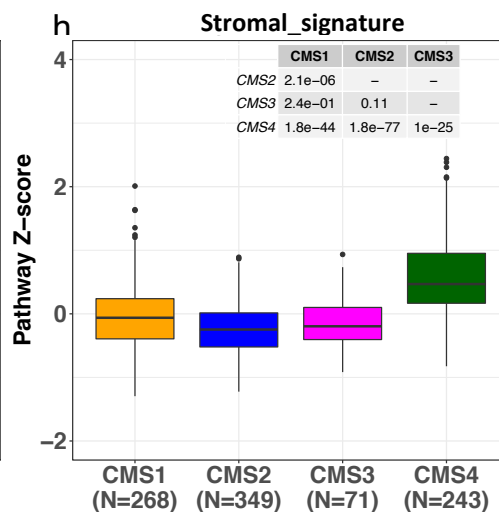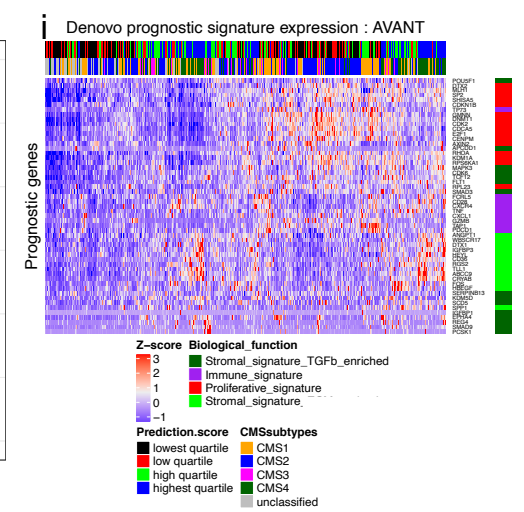

Supplement: S8 Fig — (a-d) Average expression of the four gene clusters of the AVANT signature from Fig 1c, by the AVANT signature quartiles in the AVANT BEP dataset. (e-h) Average expression of the four gene clusters of the AVANT signature by CMS subtype in the AVANT BEP dataset. The table insets show p-values computed using the pairwise T-test for each comparison adjusted for multiplicity testing. (i) Expression of the AVANT signature genes (rows) in the AVANT BEP dataset (columns). Red denotes high expression and blue denotes low expression. Patients are annotated by CMS subtype and the quartile of the AVANT signature. Row annotation indicates the respective signatures from Fig 1c. (PDF) [file pone.0262198.s008.pdf]

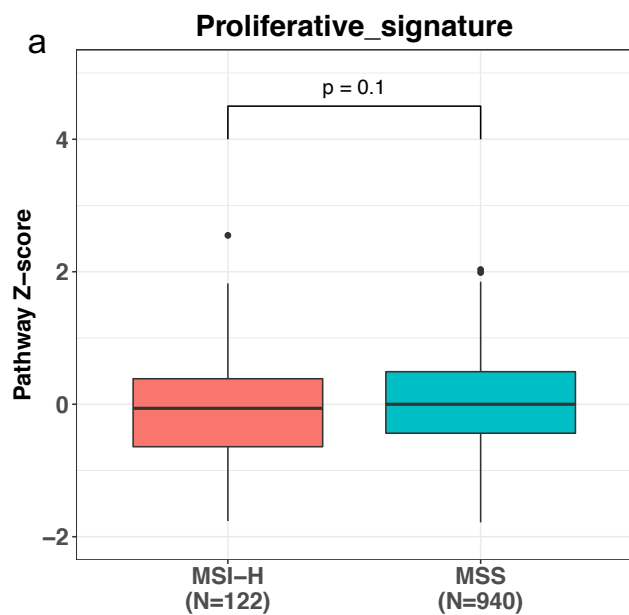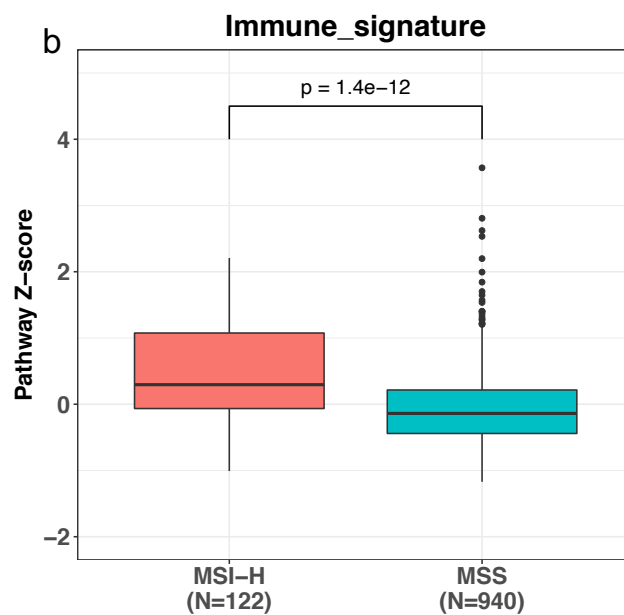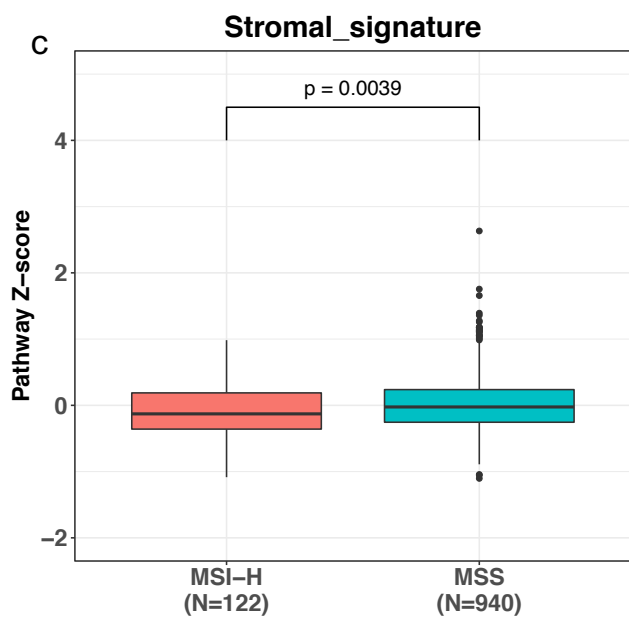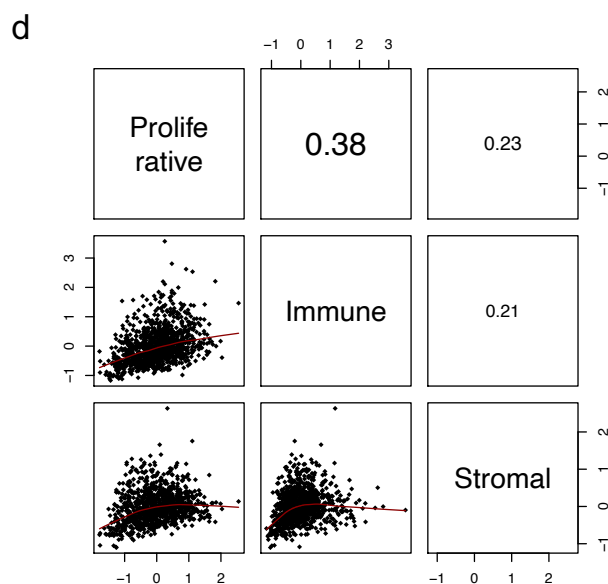

Supplement: S9 Fig — (a-c) Average expression of the gene clusters of the AVANT signature from Fig 1c by MSI status in the AVANT BEP dataset. The two stromal gene clusters were combined for panel (c). P-values correspond to a T-test. (d) Correlation between the average expression of the gene clusters of the AVANT signature in the AVANT BEP dataset, with the two stromal gene clusters combined. Pearson correlations are denoted, with font size reflecting significance. (PDF) [file pone.0262198.s009.pdf]

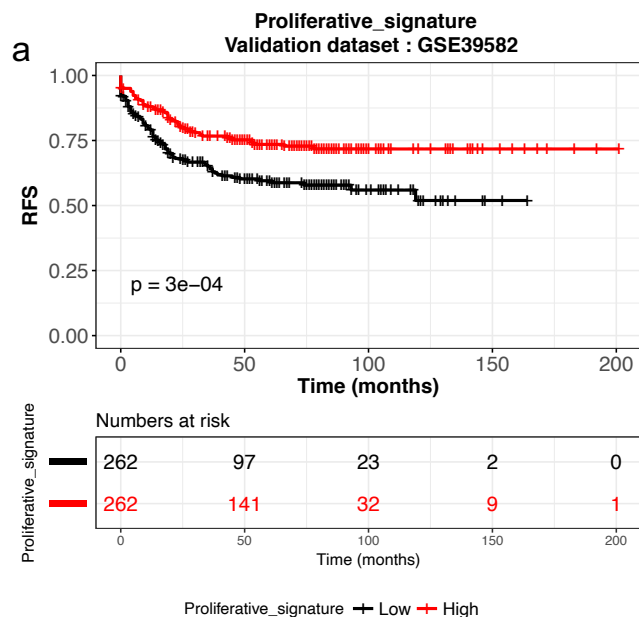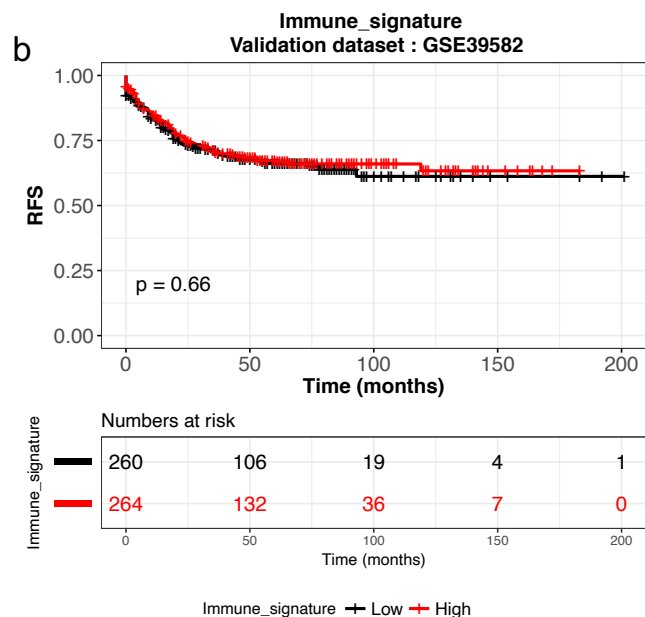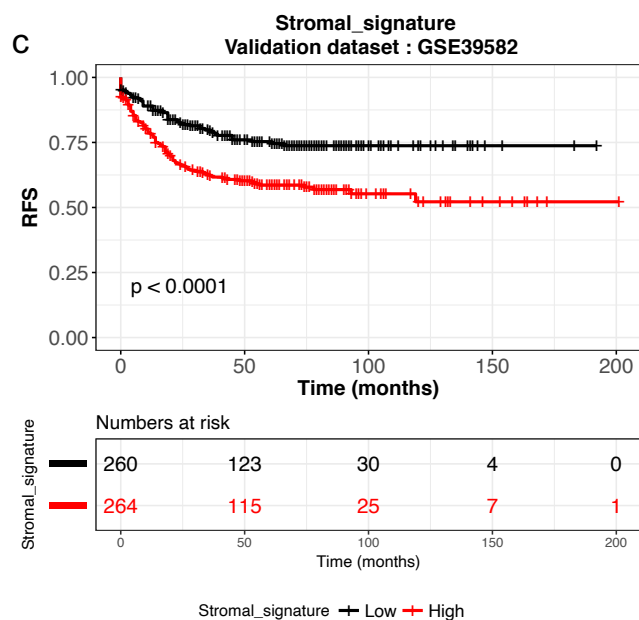

Supplement: S10 Fig — (a-c) Kaplan-Meier curves for the gene clusters of the AVANT signature from Fig 1c (combining the two stromal gene clusters) stratified by median, in the GSE39582 dataset for RFS. P-values correspond to a log-rank test. (PDF) [file pone.0262198.s010.pdf]

**GZMB**  
**Validation set: GSE39582 (CMS2, n=177)**

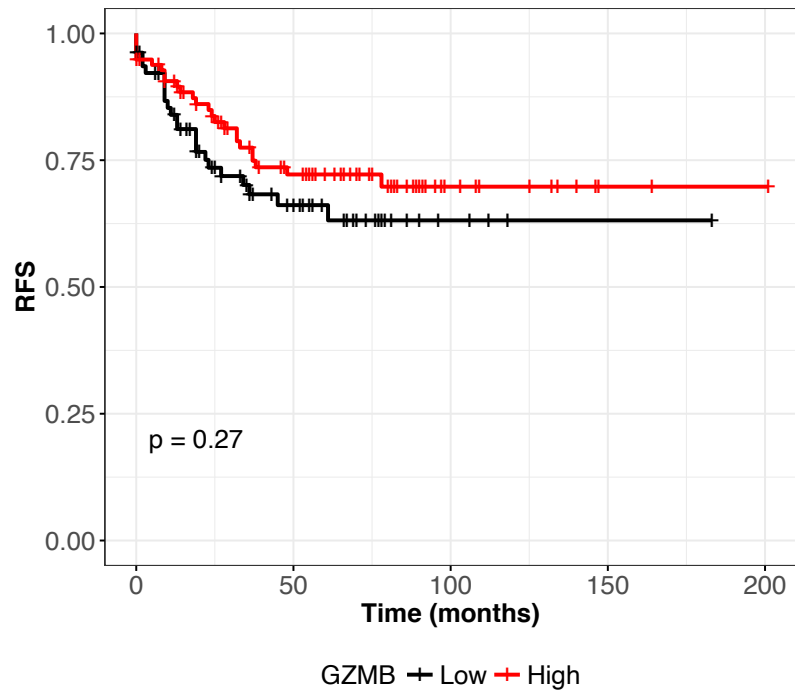

Supplement: S13 Fig — A T-effector score, excluding GZMB, below the mean score in the full cohort is considered low. The p-value corresponds to a log-rank test. (PDF) [file pone.0262198.s013.pdf]

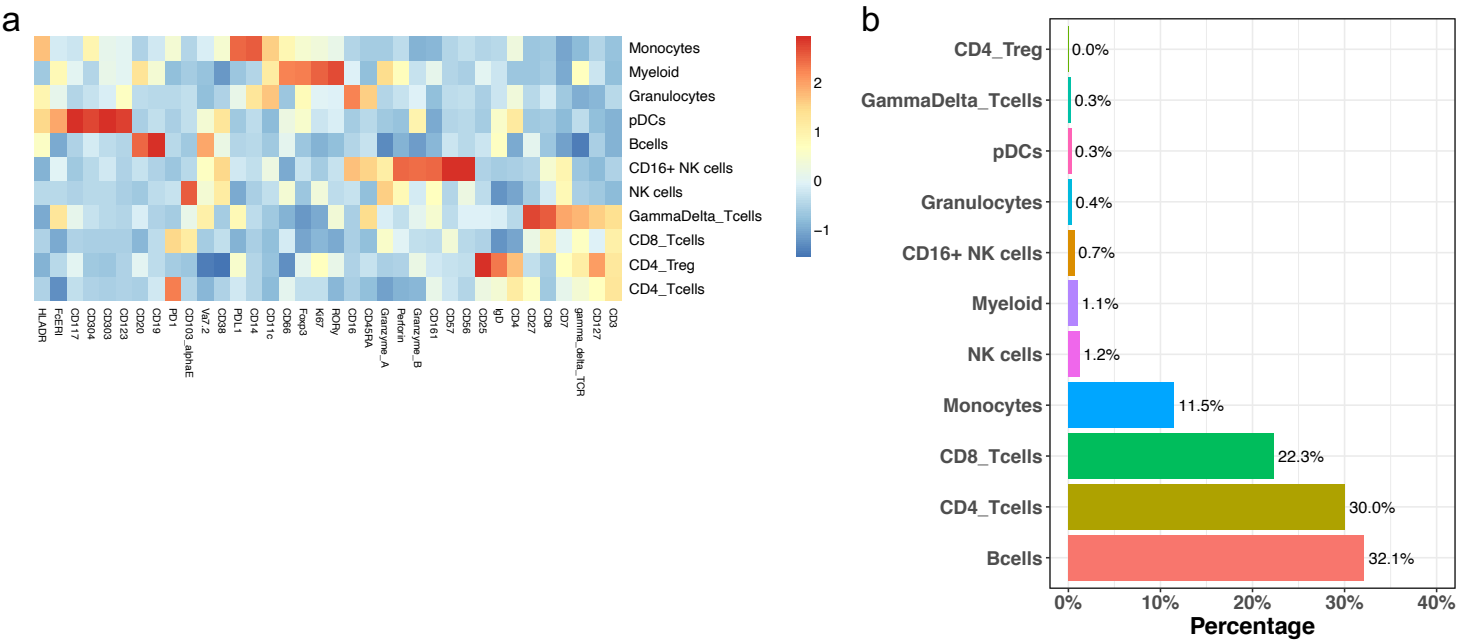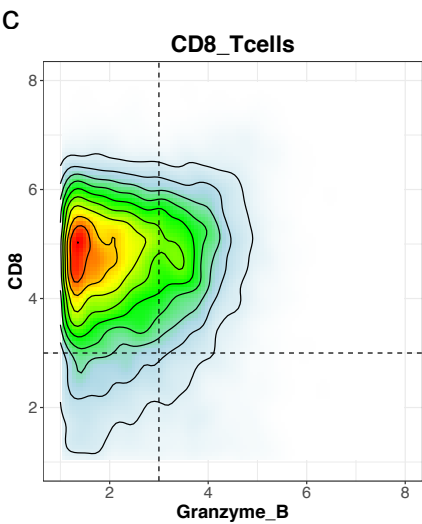

Supplement: S14 Fig — (a) Average expression of each marker across the detected immune cell clusters from Fig 4a. Shown are row scaled arcsinh transformed intensity values. Red denotes high expression; blue denotes low expression. (b) Percentage of different immune cell populations across the 12 CRC patients. (c) CD8 versus GZMB expression in CD8+ T cells. (d) Pearson correlation between expression of single genes from the pDC signature and the average expression of the other pDC genes without the gene in question, by cancer type and, for colon, by CMS subtype, in TCGA data. (PDF) [file pone.0262198.s014.pdf]

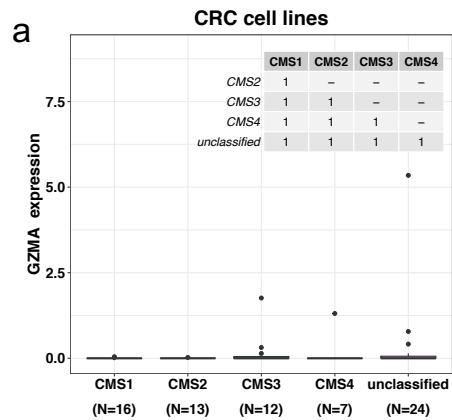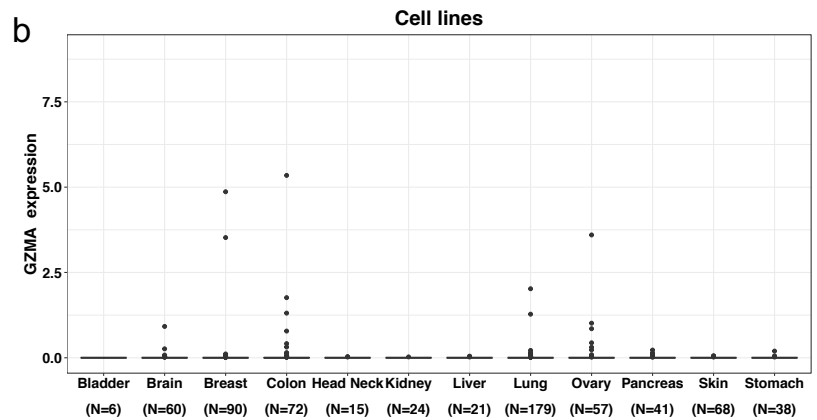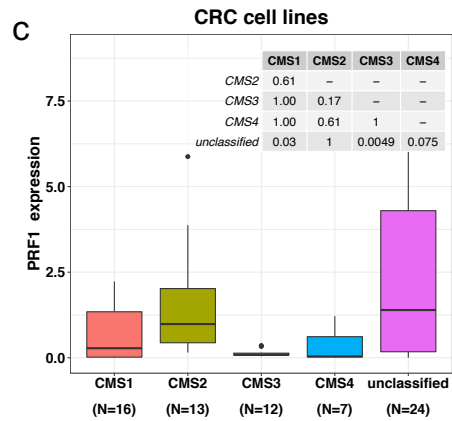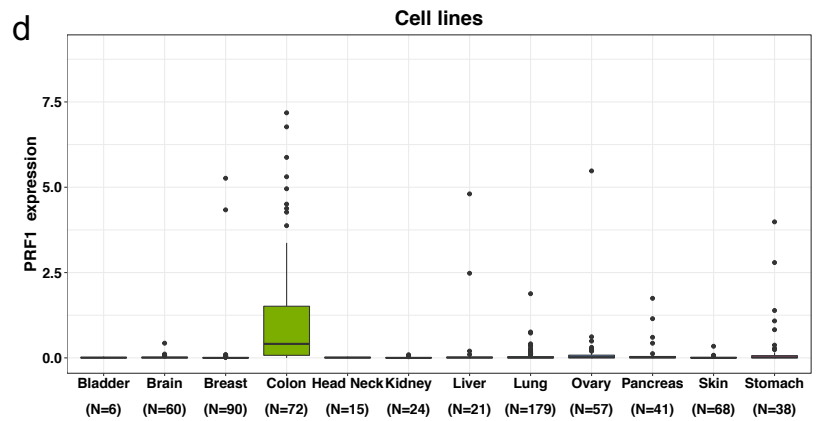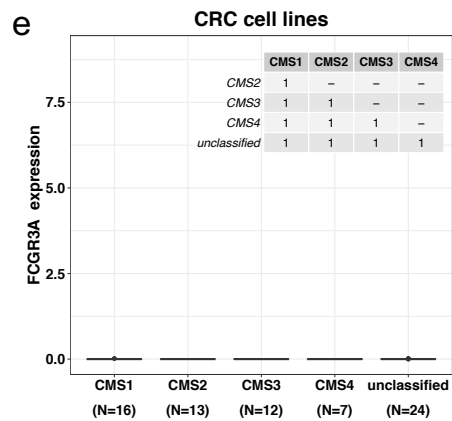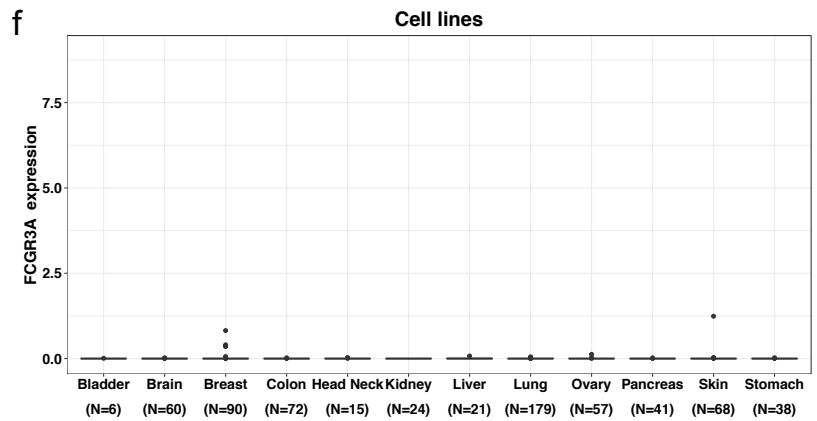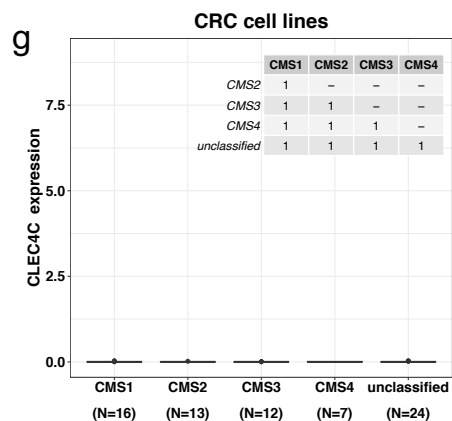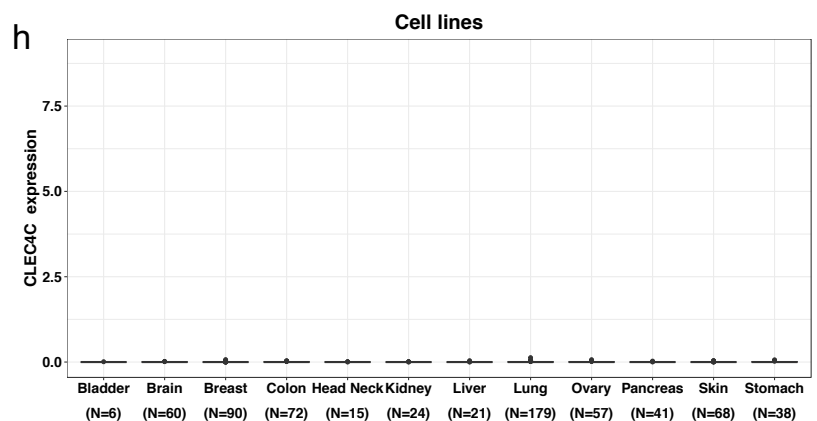

Supplement: S15 Fig — (a-b) Expression (log2 nRPKM+1) of T-effector marker GZMA in (a) CRC cell lines by CMS subtype and (b) a cohort of 671 cell lines covering 12 cancer types. (c-d) Expression (log2 nRPKM+1) of T-effector marker PRF1 in (c) CRC cell lines by CMS subtype and (d) a cohort of 671 cell lines covering 12 cancer types. (e-f) Expression (log2 nRPKM+1) of NK cell marker CD16 (FCGR3A) in (e) CRC cell lines by CMS subtype and (f) a cohort of 671 cell lines covering 12 cancer types. (g-h) Expression (log2 nRPKM+1) of pDC cell marker CLEC4C in (g) CRC cell lines by CMS subtype and (h) a cohort of 671 cell lines covering 12 cancer types. (PDF) [file pone.0262198.s015.pdf]
