# Supplementary material for: Transcriptomic profiling of adjuvant colorectal cancer identifies three key prognostic biological processes and a disease specific role for granzyme B
Source: PLoS One. 2021 Dec 31;16(12):e0262198. doi: 10.1371/journal.pone.0262198 (PMC8719661; doi:10.1371/journal.pone.0262198)
Supplement: S7 Fig — (a) Kaplan-Meier curves for the AVANT signature with use of weighted fitted coefficients in an independent validation cohort GSE39582 for RFS. (b) RFS forest plot for validation dataset GSE39582 shows hazard ratios and associated p-values for each individual signature, with use of weighted fitted coefficients for the AVANT signature as in panel (a). In the table below for GSE39582, column (1) assesses significance of added prognostic value (if any) provided by each published signature when added to the AVANT signature; column (2) assesses significance of added prognostic value provided by the AVANT signature when added to each of the individual published signatures. (PDF) [file pone.0262198.s007.pdf]

a

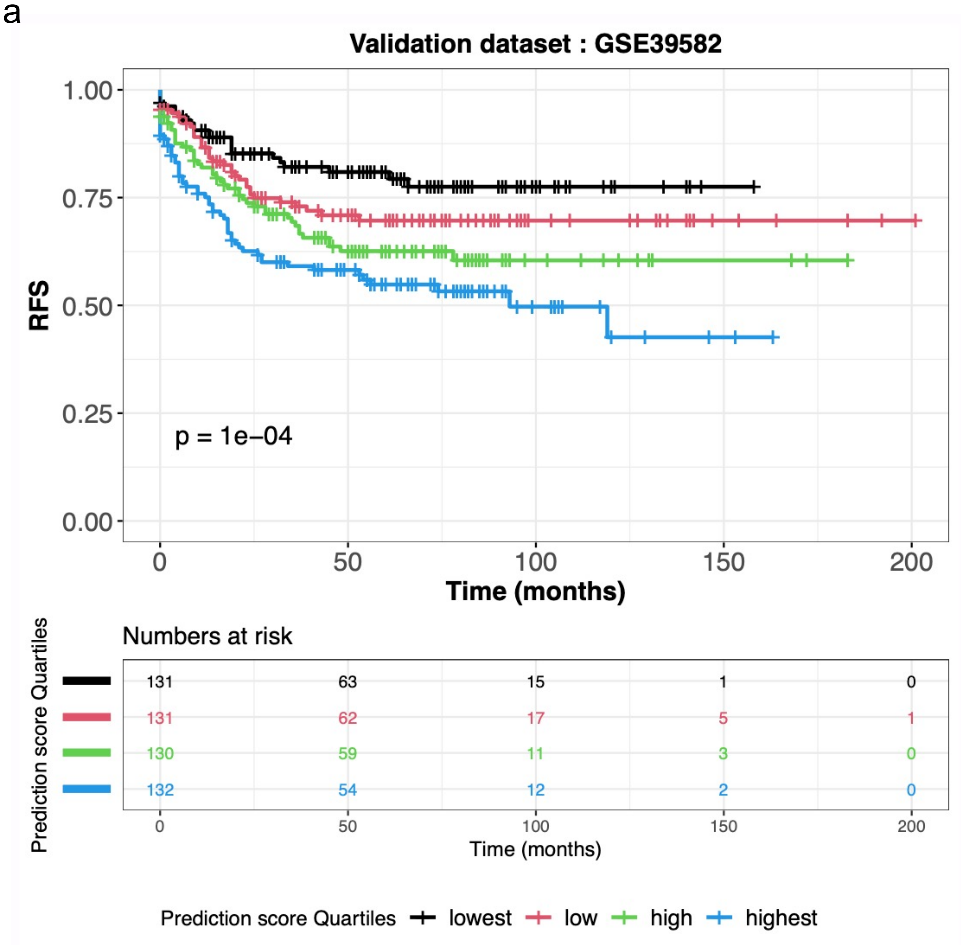

b

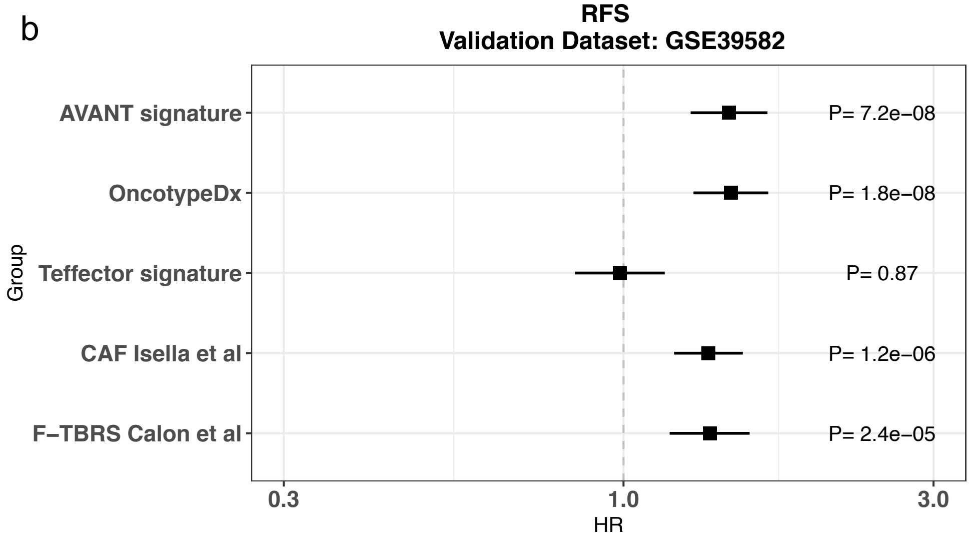

Group	Published signature added benefit to AVANT signature (P−value)	AVANT signature added benefit to published signatures (P−value)
OncotypeDx	0.017	3.5e−02
Teffector signature	0.700	4.0e−07
CAF Isella et al	0.230	5.8e−03
F−TBRS Calon et al	0.700	1.5e−03
CMS subtypes	0.016	4.7e−04
