# Supplementary material for: Transcriptomic profiling of adjuvant colorectal cancer identifies three key prognostic biological processes and a disease specific role for granzyme B
Source: PLoS One. 2021 Dec 31;16(12):e0262198. doi: 10.1371/journal.pone.0262198 (PMC8719661; doi:10.1371/journal.pone.0262198)
Supplement: S11 Fig — The table below indicates the significance of added prognostic value (if any) provided by each published signature when added to the AVANT signature (first column) and vice versa (second column). (PDF) [file pone.0262198.s011.pdf]

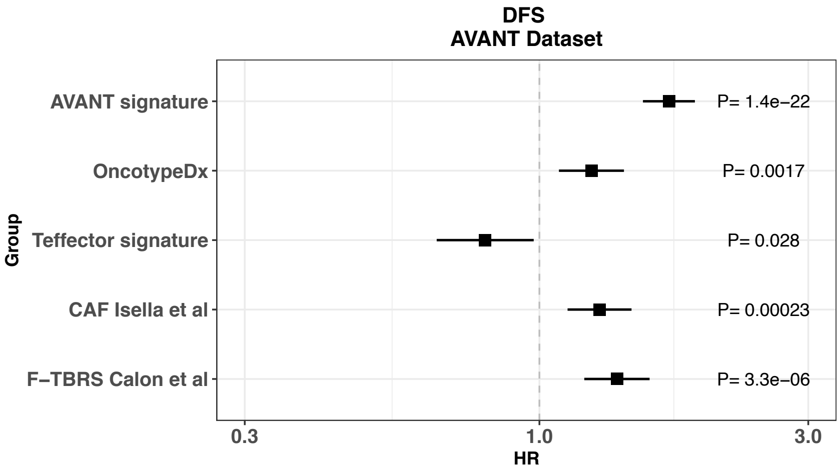

Group	**Published Signature added benefit to AVANT signature (P−value)**	**AVANT signature added benefit to published signatures (P−value)**
OncotypeDx	0.064	2.7e−16
Teffector signature	0.059	4.6e−17
CAF Isella et al	0.010	2.9e−16
F−TBRS Calon et al	0.130	1.2e−13
CMS subtypes	0.010	1.3e−04
