# Supplementary material for: Transcriptomic profiling of adjuvant colorectal cancer identifies three key prognostic biological processes and a disease specific role for granzyme B
Source: PLoS One. 2021 Dec 31;16(12):e0262198. doi: 10.1371/journal.pone.0262198 (PMC8719661; doi:10.1371/journal.pone.0262198)
Supplement: S12 Fig — Forest plots denote the Cox hazard ratios (HR) and p-values for the different signature quartiles after adjustment for clinical covariates. Each individual clinical covariate in each trial was tested for its effect on prognosis (DFS or RFS) and only prognostic covariates were included in the multivariate analyses. (a) Included covariates in the AVANT BEP dataset were age, sex, level of CEA in blood, ECOG status and AJCC tumor status including lymph node status (i.e. strata). (b) Included covariates in the GSE39582 dataset were age, sex, tumor stage, and lymph node status. (PDF) [file pone.0262198.s012.pdf]

a

Association of AVANT signature with DFS after adjustment for covariates AVANT dataset

Cutoff (%)	Cutoff value	Low Events/N	High Events/N	HR (95%CI)	P
25%	−0.35	24/265	159/797	2.15 (1.39–3.33)	0.00055
50%	−0.08	62/531	121/531	1.86 (1.36–2.54)	9.5e−05
75%	0.25	100/796	83/266	2.61 (1.94–3.52)	2.5e−10

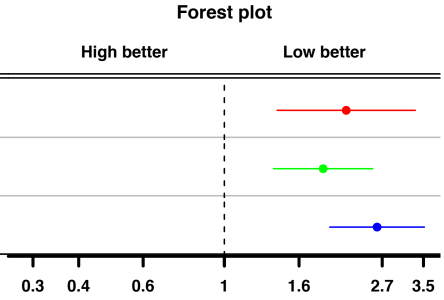

Results adjusted by
AGE_CAT , CEABL , ECOGSCAT , SEX , STRATA

b

Association of AVANT signature with RFS after adjustment for covariates GSE39582 dataset

Cutoff (%)	Cutoff value	Low Events/N	High Events/N	HR (95%CI)	P
25%	−0.31	24/131	140/393	1.95 (1.22–3.12)	0.0052
50%	−0.08	60/262	104/262	1.93 (1.36–2.73)	0.00021
75%	0.24	106/392	58/132	1.64 (1.16–2.32)	0.005

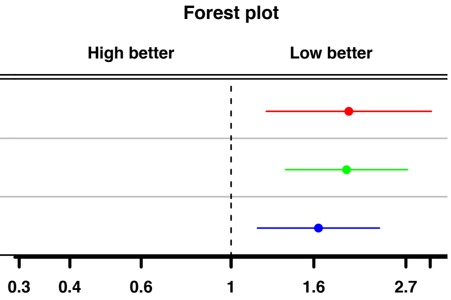

Results adjusted by
age.at.diagnosis , tnm.stage , tnm.n , Sex
