# Supplementary material for: Transcriptomic profiling of adjuvant colorectal cancer identifies three key prognostic biological processes and a disease specific role for granzyme B
Source: PLoS One. 2021 Dec 31;16(12):e0262198. doi: 10.1371/journal.pone.0262198 (PMC8719661; doi:10.1371/journal.pone.0262198)
Supplement: S4 Table — (PDF) [file pone.0262198.s019.pdf]

Supplemental Table 4. Overview of clones and vendors of a 37 parameter isotope conjugated panel of monoclonal antibodies for lineage and functional markers of immune populations.

Mass	Metal	Target	Source	Vendor	Clone
89	Y	CD45	Fluidigm		HI30
113	In	CD57	Custom	Biolegend	HCD57
115	In	EpCAM	Custom	Biolegend	9C4
139	La				
140	Ce	EQ Beads			
141	Pr				
142	Nd	CD19	Fluidigm		HIB19
143	Nd				
144	Nd	Granzyme B	Custom	Biolegend	GB11
145	Nd	CD4	Fluidigm		RPA-T4
146	Nd	IgD	Fluidigm		IA6-2
147	Sm	CD7	Fluidigm		CD7-6B7
148	Nd	RORγT	Custom	eBioscience	AFKJS-9
149	Sm	Granzyme A	Custom	Biolegend	CB9
150	Nd	CD66	Custom	BD	B1.1
151	Eu	CD123	Fluidigm		6H6
152	Sm	TCR	Custom	Life Technologies	SA6.E9
153	Eu	CD117	Custom	Biolegend	104D2
154	Sm	CD3	Fluidigm		UCHT1
155	Gd	CD27	Fluidigm		L128
156	Gd	CD304	Custom	Biolegend	12C2
157	Gd	CD38	Custom	Biolegend	HIT2
158	Gd	Vα7.2	Custom	Biolegend	3C10
159	Tb	CD11c	Fluidigm		Bu15
160	Gd	CD303	Custom	Biolegend	201A
161	Dy	Ki67	Fluidigm		B56
162	Dy	Foxp3	Fluidigm		PCH101
163	Dy	CD20	Custom	Biolegend	2H7
164	Du	CD161	Fluidigm		HP-3G10
165	Ho	CD127	Fluidigm		A019D5
166	Er	FcεRI	Custom	Biolegend	AER-37 (CRA-1)
167	Er	CD103	Custom	Biolegend	Ber-ACT8
168	Er	CD8	Fluidigm		SK1
169	Tm	CD25	Fluidigm		2A3
170	Er	CD45RA	Fluidigm		HI100
171	Yb	PDL1	Custom	Genentech	14D3
172	Yb	CD14	Custom	Biolegend	M5E2
173	Yb	PD-1	Custom	Biolegend	EH12.2H7
174	Yb	HLA-DR	Fluidigm		L243
175	Lu	Perforin	Fluidigm		B-D48
176	Yb	CD56	Custom	Miltenyi	REA196
191	Ir	Nucleic acid	Fluidigm		
192	Pt	Cisplatin	Fluidigm		
193	Ir	Nucleic acid	Fluidigm		
195	Pt	Cisplatin	Fluidigm		
209	Bi	CD16	Fluidigm		3G8

**Fluorescence**	**Target**	**Vendor**	**Clone**
FITC	CD3	Biolegend	UCHT1
FITC	CD14	Biolegend	M5E2
FITC	CD20	Biolegend	2H7
AF647	Granzyme B	Biolegend	GB11
PE-Cy7	HLA-DR	Biolegend	L243
BV421	CD123	Biolegend	6H6
APC-H7	Live/Dead	Biolegend	
