# Supplementary material for: Transcriptomic profiling of adjuvant colorectal cancer identifies three key prognostic biological processes and a disease specific role for granzyme B
Source: PLoS One. 2021 Dec 31;16(12):e0262198. doi: 10.1371/journal.pone.0262198 (PMC8719661; doi:10.1371/journal.pone.0262198)
Supplement: S5 Table — (PDF) [file pone.0262198.s020.pdf]

Supplemental Table 5. Clinical characteristics in the Intent-to-Treat Population by treatment arm and the Biomarker Evaluable Population**.**

	**Intent-to-Treat Population**			**Biomarker Evaluable Population[a]**
	**FOLFOX4[c] (N=1151)**	**Bevacizumab plus FOLFOX4 (N=1155)**	**Bevacizumab plus XELOX (N=1145)**	
**Disease Stage**				
Stage II (high-risk)	192 (17%)	194 (17%)	187 (16%)	182 (17.1%)
Stage III	955 (83%)	960 (83%)	952 (83%)	880 (82.8%)
Stage III - N1	585 (51%)	590 (51%)	572 (50%)	576 (54.2%)
Stage III - N2	370 (32%)	370 (32%)	380 (33%)	304 (28.6%)
**Age**				
Mean (years)	58	58	58	59
**Sex**				
Men	656 (57%)	587 (51%)	625 (55%)	588 (55.4%)
Women	495 (43%)	568 (49%)	520 (45%)	474 (44.6%)
**ECOG[b] Performance Status**				
0	994 (86%)	987 (85%)	978 (85%)	922 (86.9%)
1	156 (14%)	166 (14%)	165 (14%)	139 (13.1%)
**Ethnic Origin**				
White	956 (83%)	976 (85%)	963 (84%)	999 (94.01%)
Asian	158 (14%)	138 (12%)	138 (12%)	63 (5.9%)
**Treatment Arm**				
FOLFOX	1151	0	0	343 (32.3%)
Bevacizumab plus FOLFOX4	0	1155	0	366 (34.5%)
Bevacizumab plus XELOX	0	0	1145	353 (33.2%)

a: Analysis has been conducted on all treatment arms jointly
b: ECOG- Eastern Co-operative Oncology Group.
c: fluorouracil, leucovorin, and oxaliplatin
d: capecitabine (Xeloda) plus oxaliplatin
Patient numbers across disease stage do not add up to total Intent-to-Treat Population since 11 patients had diseases across stages (I, II, IV).
